# Histological characteristics of exercise‐induced skeletal muscle remodelling

**DOI:** 10.1111/jcmm.17879

**Published:** 2023-07-30

**Authors:** Qihang Su, Jie Li, Jingbiao Huang, Qiuchen Cai, Chao Xue, Chenglong Huang, Liyang Chen, Jun Li, Dandan Li, Hengan Ge, Biao Cheng

**Affiliations:** ^1^ Department of Sports Medicine, Tongji Hospital, School of Medicine Tongji University Shanghai China; ^2^ Department of Orthopedics, Shanghai Tenth People's Hospital, School of Medicine Tongji University Shanghai China; ^3^ Department of Orthopedics Zhabei Central Hospital of Jing'an District Shanghai China; ^4^ Department of Medical Ultrasound, Shanghai Tenth People's Hospital, Ultrasound Research and Education Institute, Shanghai Engineering Research Center of Ultrasound Diagnosis and Treatment, School of Medicine Tongji University Shanghai China; ^5^ Department of Environmental and Public Health Sciences, College of Medicine University of Cincinnati Cincinnati Ohio USA

**Keywords:** angiogenesis, exercise‐induced muscle injury, load exercise, skeletal muscle remodelling, temporal regulation

## Abstract

This study aims to analyse the pathological features of skeletal muscle injury repair by using rats to model responses to different exercise intensities. Eighty‐four rats were randomly divided into five groups for treadmill exercise. The short‐term control, low‐intensity, medium‐intensity and high‐intensity groups underwent gastrocnemius muscle sampling after 6, 8 and 12 weeks of exercise. The long‐term high‐intensity group underwent optical coherence tomography angiography and sampling after 18 weeks of exercise. RNA sequencing was performed on the muscle samples, followed by the corresponding histological staining. Differentially expressed genes were generally elevated at 6 weeks in the early exercise stage, followed by a decreasing trend. Meanwhile, the study demonstrated a negative correlation between time and the gene modules involved in vascular regulation. The modules associated with muscle remodelling were positively correlated with exercise intensity. Although the expression of many genes associated with common angiogenesis was downregulated at 8, 12 and 18 weeks, we found that muscle tissue microvessels were still increased, which may be closely associated with elevated sFRP2 and YAP1. During muscle injury‐remodelling, angiogenesis is characterized by significant exercise time and exercise intensity dependence. We find significant differences in the spatial distribution of angiogenesis during muscle injury‐remodelling, which be helpful for the future achievement of spatially targeted treatments for exercise‐induced muscle injuries.

## 1 INTRODUCTION

Exercise‐induced muscle injuries (EIMIs) are prevalent in sports involving high‐speed running or high volumes of running load, acceleration, deceleration and upon fatiguing conditions of play or performance.^1^ Among them, calf muscle injuries are common in sports involving high‐speed running, explosive jumping, and kicking. The calf complex is an essential body structure for weight bearing and locomotive activity. Skeletal muscular dysfunction, pain and oedema are the major presenting characteristics of calf muscle injuries.^2^ The duration of rehabilitation until return to regular sports is usually quite lengthy, especially for athletes with significant injuries.

The pathological features of EIMI mainly involve muscle fibre rupture and skeletal muscle remodelling, including the remodelling of the extracellular matrix, myofibre and vascular bed.^3^ Myofibre rupture, microvessel damage and inflammatory infiltration in the early stages of injury can induce tissue regeneration and repair mechanisms.^4^, ^5^ However, the course of repair is prolonged with an uncertain prognosis. If the injury exceeds the capacity of the tissue to self‐repair, pathological muscle healing and irreversible damage can emerge, including chronic inflammation, muscular fibrosis, heterotopic ossification and muscle atrophy or stiffness.^6^, ^7^ These outcomes can have a particularly negative impact on athletes. Unfortunately, there is no optimal treatment and rehabilitation programme for EIMI. Symptomatic treatments, physical therapy and mild rehabilitative exercises often cannot fully correct the injury resulting in a suboptimal outcome.^8^ Therefore, it is necessary to understand the temporal pathological characteristics of skeletal muscle tissue under different exercise loads and cycles to identify precise treatment choices and suitable intervention times. However, few studies have addressed these topics.^9^

Physiopathological features of skeletal muscle exhibits remarkable heterogeneity and dynamic changes during exercise or injury repair. The heterogeneity and dynamics of muscle fibres are fundamental to a muscle's ability to perform a variety of tasks ranging from continuous low‐intensity activity (such as maintaining posture) to repetitive submaximal contractions (such as during locomotion) and rapid and intense maximal contractions (such as during jumping and kicking).^10^ Currently published studies show that many cytokines or signalling pathways are favourable for EIMI repair and thus have marked potential for clinical translation.^11^, ^12^ A better understanding of exercise‐dependent muscle change can help identify potential therapeutic targets.

We hypothesized that the histological and transcriptomic characteristics of skeletal muscle were distinctly different and highly heterogeneous in response to time and exercise intensity. In this study, we explored the histological and transcriptomic characteristics of temporal changes in the rat gastrocnemius muscle in response to treadmill exercise of different intensities and cycles. We analysed the effect of exercise‐induced angiogenesis on skeletal muscle remodelling and the regulation of the FHL2/sFRP2 signalling axis. The study helps uncover the dynamic pathological characteristics of skeletal muscle remodelling, provides new references for molecular mechanisms of EIMI and helps in the development of clinical intervention programmes from the perspective of angiogenesis.

## 2 MATERIALS AND METHODS

### 2.1 Rats

Twelve‐week‐old male specific pathogen‐free Sprague Dawley rats weighing 250–300 g were used (Shanghai Jihui Laboratory Animal Care Co., Ltd.; *n* = 86). Rats were housed under controlled conditions (22°C, 12 h light/12 h dark cycle) with ad libitum access to water and standard laboratory rat chow. One rat died during the experiment due to exercise fatigue. One additional rat was withdrawn from the experiment after refusing to exercise continuously. At the end of the intervention, the animals were anaesthetised with 1.25% Avertin (10 mL/kg) and euthanized by cervical dislocation. Surgical interventions, treatments and animal care procedures were performed strictly with a protocol approved by the Animal Care and Use Committee of the University School of Medicine. Exclusion criteria for rats: 1. death or trauma during the experiment, for example, broken nail causing severe bleeding in the toe; 2. inability to tolerate the exercise intensity set in the exercise protocol or refusal to exercise; 3. severe physiological reactions or other conditions that affect daily life after exercise, for example, prolonged refusal to eat.

### 2.2 Exercise intervention

**FIGURE 1:**
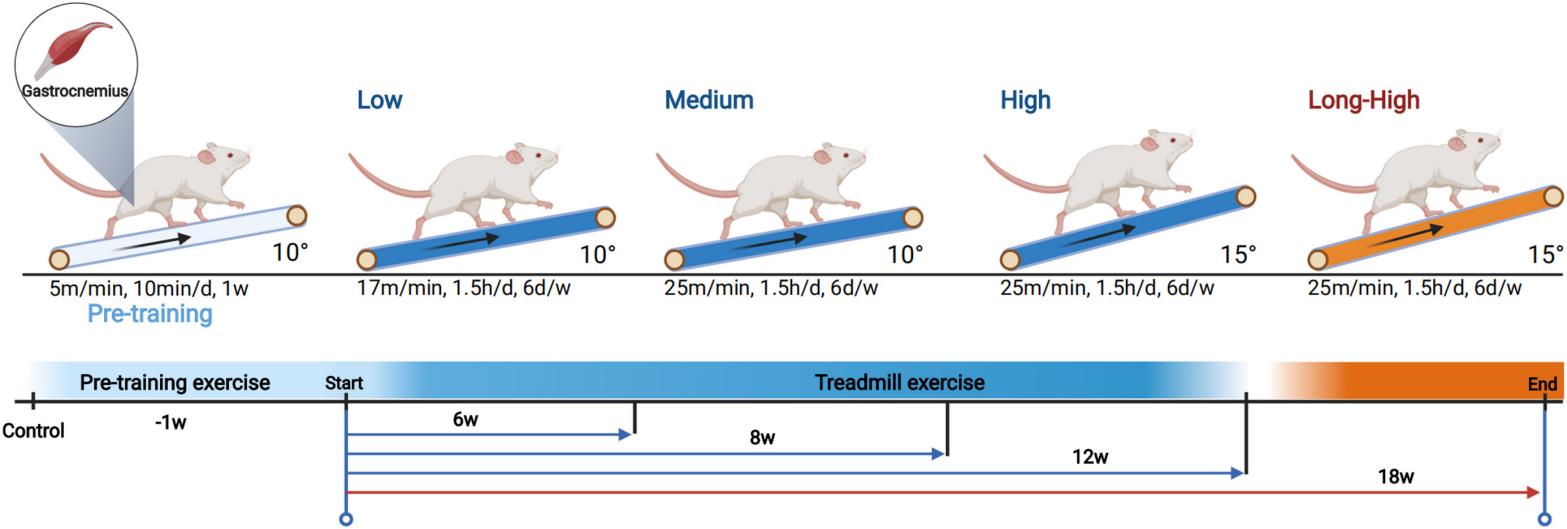
Rat exercise protocol (Created with BioRender.com). Rats were divided into control, low, medium, high and long‐term high‐intensity groups. All rats underwent adaptive pretraining except for the controls, followed by treadmill exercise training per the predetermined procedures. The low, medium and high‐intensity groups underwent training for 6, 8 and 12 weeks. The long‐term high‐intensity group was trained for 18 weeks.

Firstly, we determined the maximum tolerance and minimum threshold for muscle damage in rats using previous literature,^13^, ^14^, ^15^, ^16^ then developed a gradient training protocol within this range and finally clarified the feasibility of the training protocol through pre‐experiments and haematoxylin and eosin staining to maximize the simulation of the clinical EIMI disease state by active exercise. Eighty‐four rats were randomly divided into five groups for treadmill exercise (*n* = 6, per time point, per group), including control (no treadmill exercise), low‐intensity, medium‐intensity, high‐intensity and long‐term high‐intensity groups. All rats underwent adaptive pretraining except for the controls, with 5 m/min speed settings at 10° uphill for 10 min daily for 1 week. Rats were trained at varying load intensities according to the designed speed, time and treadmill angle (Figure 1). Low ‐intensity was 17 m/min speed, 10° uphill, 1.5 h of cumulative daily training (30 min of rest every half hour of exercise to provide food and water) and 6 days per week for 6, 8 and 12 weeks. Medium intensity was 25 m/min speed, 10° uphill, 1.5 h of cumulative daily training (30 min of rest every half hour of exercise to provide food and water) and 6 days per week for 6, 8 and 12 weeks. High ‐intensity was 25 m/min speed, 15° uphill, 1.5 h of cumulative daily training (30 min of rest every half hour of exercise to provide food and water), and 6 days per week for 6, 8 and 12 weeks. The long‐term high‐intensity group condition was 25 m/min speed, 15° uphill, 1.5 h of cumulative daily training (30 min of rest every half hour to provide food and water) and 6 days per week for 18 weeks. All exercise group rats were motivated to run with a shock grid set at 0.4 mA. At each sampling time point (6, 8, 12 and 18 weeks) six rats were randomly selected from each group for analysis.

### 2.3 Muscle samples

Rats were intraperitoneally administered 1.25% Avertin (10 mL/kg) (Nanjing Aibei Biotechnology Co., Ltd) to induce anaesthesia and euthanized by cervical dislocation 3 days after the relevant exercise protocol was completed.^17^ Gastrocnemius muscle tissue was obtained surgically. A portion of gastrocnemius muscle tissue was cryopreserved in liquid nitrogen and used for RNA sequencing (RNA‐seq). The remaining tissue was fixed in 4% paraformaldehyde and embedded in paraffin. Four‐micrometre sections were deparaffinized and mounted on glass slides at room temperature (pathology slicer and Leica embedder provided by Shanghai Leica Instrument Co., Ltd.) until staining. We mainly chose the larger midsection of the muscle for our sections: coronal sections for muscle fibre type analysis and longitudinal sections for other histological analyses. Six microscopic views at the same magnification will be selected for analysis for each tissue section, and the mean or median will be calculated.

### 2.4 RNA sequencing

Four rats (six from the long‐term high‐intensity group) were randomly selected from each group from each time point. Total RNA was extracted from fresh gastrocnemius muscle tissues using an RNeasy Mini Kit (Cat#74106, Qiagen). Agilent Bioanalyzer 4200 (Agilent Technologies) was used to detect RNA quality. Sequencing libraries were prepared using the VAHTSTM Stranded mRNA‐seq Library Prep Kit (NR612, Vazyme). The cDNA library was sequenced through the Illumina sequencing platform (Novaseq). The RNA isolation, library construction and sequencing were performed at Shanghai Biochip Co. Ltd. Differentially expressed genes (DEGs) were identified according to *Q* < 0.05 and |log_2_(fold change)| ≥2. Gene Ontology (GO) terms and Kyoto Encyclopedia of Genes and Genomes (KEGG) pathways with *Q* < 0.05 were considered to be significantly enriched. Data visualization with volcano plots was performed using Hiplot software (https://hiplot.com.cn) (*p* < 0.05 and |log_2_(fold change)| ≥0.5). Heat maps were created using the R package clusterProfiler. R software was used to conduct the weighted gene co‐expression network analysis (WGCNA) (Time; Exact intensity).

We screened for all DEGs, and then calculated the average expressions of differential genes using the corresponding FPKM values at each time point. A time‐series gene cluster analysis was performed on gene expression data using the R package Mfuzz (2.52.0) to identify the clusters with consistent expression trends.

### 2.5 Histological staining

The prepared muscle sections were stained with Haematoxylin and Eosin, periodic acid‐schiff (PAS), Masson's trichrome (Masson) and sirius red (SR) following routine procedures.^17^ Microscope images were obtained at different magnifications using a scanning imaging system (ECLIPSE E100 and DS‐U3, NIKON). The Supplementary Methods file shows the method details.

### 2.6 Optical coherence tomography angiography (OCTA)

OCTA (LSM02/03, spectral bandwidth 100 nm, central wavelength 1310 nm, transverse image resolution 15 μm, Beijing HealthOLight Technology Co., Ltd) was used to assess the intravital vessels in the control and long‐term high‐intensity groups. Live rats were anaesthetised and shaved over the gastrocnemius muscle region of the hind limbs. Vascular proliferation and distribution were assessed directly using the OCT system.

### 2.7 Scratch wound healing assay

Human umbilical vein endothelial cells (HUVECs) (iCell Bioscience Inc, Shanghai; 5 × 10^5^ cells/mL) were seeded in six‐well plates (10% serum medium for HUVECs culture and expansion, HUVEC‐90011 with the growth factor supplementation, OriCell®) and cultured in 37°C incubators at 5% CO_2_ for 24 h. The six‐well plate was removed from the incubator after the cells grew to the logarithmic phase and the adhesion rate reached 80%–90%. The spent medium was aspirated and a 20 μL pipette tip was used to make a transverse scratch on the culture plate, with the tip remaining vertical during the procedure. Each well was manipulated in the same method. Subsequently, the cells were rinsed three times with phosphate buffered saline (PBS). Next, the scratched cells were aspirated and divided into four groups by adding 2% low serum medium (HUVEC‐90011 without the growth factor supplementation, OriCell®) with 1× PBS (Wuhan Servicebio Technology Co., Ltd.), 10 pM Recombinant human frizzled‐related protein 2 (rhsFRP2) (CSB‐MP021139HU, Cusabio Biotech),^18^ 10 nM Recombinant human Yes‐associated protein 1 (rhYAP1) (CSB‐YP026244HU, Cusabio Biotech) and 10 pM rhsFRP2^+^ 10 nM Peptide 17 (YAP‐TEAD Inhibitor 1, S8164, Selleck).^19^ The medium was stored in the incubator for 12 h before imaging (100×, the same area of the well imaged at both time points).

### 2.8 Immunofluorescence (IF)

MYH1, MYH7, Ki‐67 and TGF‐β1 were detected by IF staining. MyoD^+^Desmin, iNOS^+^CD68 and CD163^+^CD206 were assessed using double IF staining. The Supplementary Methods file showed the method details.

### 2.9 Immunohistochemistry (IHC)

IHC targets included IL‐1β, IL‐6, TNF‐α, CD34, vWF, VEGFA, sFRP2, YAP1, phospho‐YAP1^S127^ and FHL2.^17^ Vascular density was quantified by IHC results for CD34 and vWF. The Supplementary Methods file shows the method details.

### 2.10 Terminal deoxynucleotidyl transferase dUTP nick end labeling (TUNEL) assay

TUNEL assay was performed on sections utilizing conventional methods to quantify the apoptotic cell proportion.^20^ The Supplementary Methods file shows the method details.

### 2.11 Statistical analysis

All data were analysed using IBM SPSS Statistics for Windows, version 20 (IBM Corp.). Statistical significance was set at *p* < 0.05. Data from PAS, Masson, SR, IF, IHC and TUNEL were evaluated using a simple effect analysis with a factorial design (Table S1 shows a significant interaction between groups and time variables). Scratch closure ratio values were normally distributed and showed homoscedasticity and thus assessed by a one‐way anova (Table S2). All specific relevant statistical results are presented in Tables S3–S24. Spearman's rank correlation coefficient was adopted for analysis in the correlation heat map.

## 3 RESULTS

### 3.1 DEGs in gastrocnemius muscles under different exercise intensities

**FIGURE 2:**
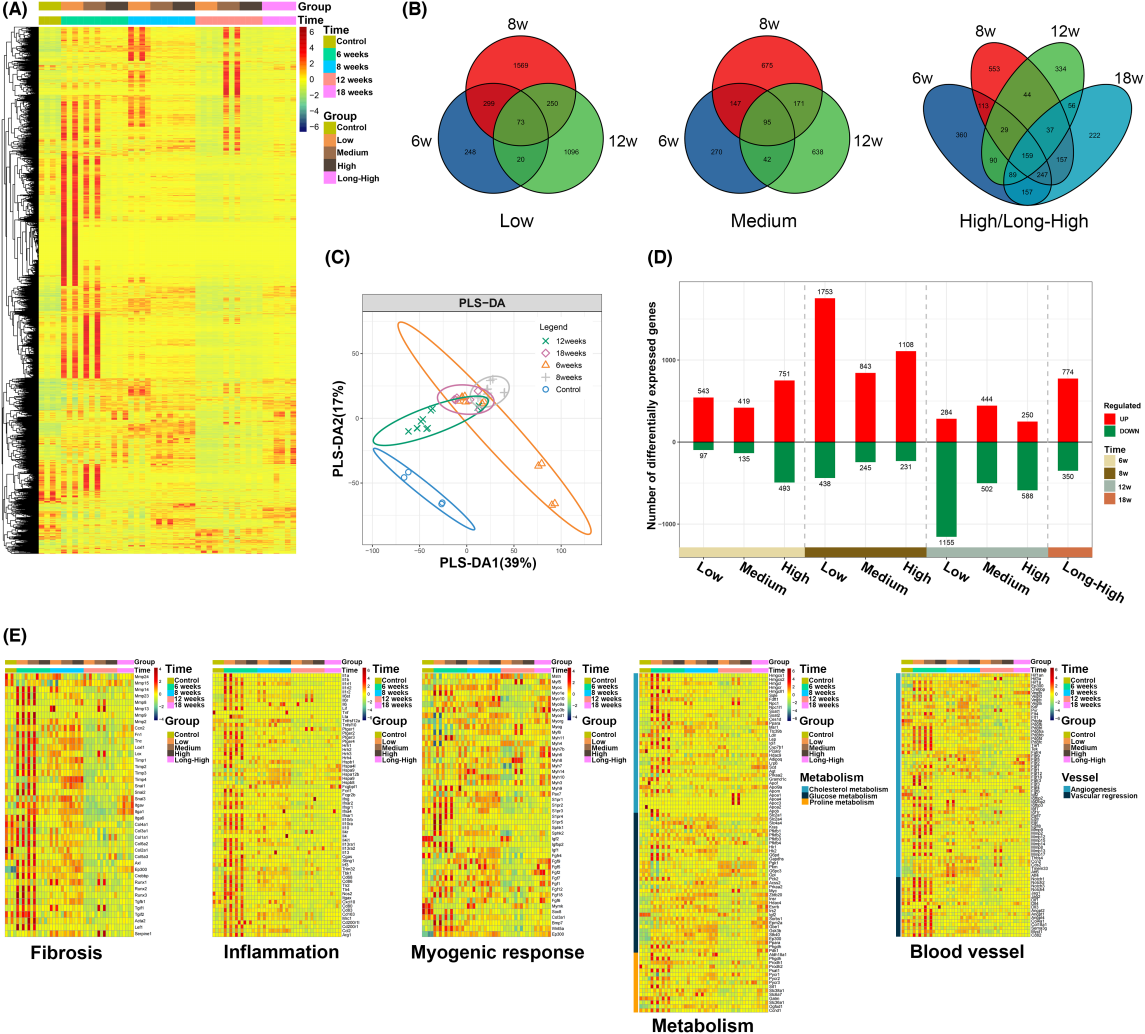
RNA‐seq analysis of rat skeletal muscle. (A) Heat map of overall gene expression; (B) venn diagram showing the intersection of differentially expressed genes in the low, medium, high and long‐term high‐intensity groups at different time points (vs. control group, *Q* < 0.05, |log_2_(fold change)| ≥2); (C) partial least squares discriminant analysis; (D) statistical plots of the number of upregulated and downregulated differentially expressed genes in the low, medium, high and long‐term high‐intensity groups (vs. control group) at different time points; (E) heat map of commonly expressed genes associated with fibrosis, inflammation, myogenic response, metabolism and angiogenesis.

The mRNA heat map demonstrated a considerable temporal fluctuation in the correlation between DEGs and exercise intensity (Figure 2A). A Venn diagram revealed that the number of DEGs showed a significant temporal change in the low, medium and long‐term high‐intensity groups, compared to the control group (*Q* < 0.05, |log_2_(fold change)| ≥2). The expression of DEGs rose from 6 to 8 weeks and decreased at 12 or 18 weeks (Figure 2B). Partial least squares discriminant analysis showed significant differences between the control group and the exercise model at 6, 8, 12 and 18 weeks (Figure 2C); However, no significant differences were shown among 6, 8, 12 and 18 weeks, which may be related to the fact that the exercise intensity variable was not considered. Significantly upregulated DEGs increased in the low, medium and high‐intensity groups at 8 weeks. Significantly downregulated DEGs decreased mainly at 12 weeks. However, upregulated DEGs dramatically increased at 18 weeks. The low‐intensity group showed an extensive change range (1753 upregulated DEGs at 8 weeks and 1155 downregulated DEGs at 12 weeks) (Figure 2D). The trend was also found in DEGs associated with fibrosis, inflammation, myogenic response, metabolism (cholesterol, glucose and proline) and vascular remodelling (Figure 2E). It is clear that the DEGs are characterized by significant time‐series variability, and we will carry out further time‐series analysis.

### 3.2 DEGs timing analysis

**FIGURE 3:**
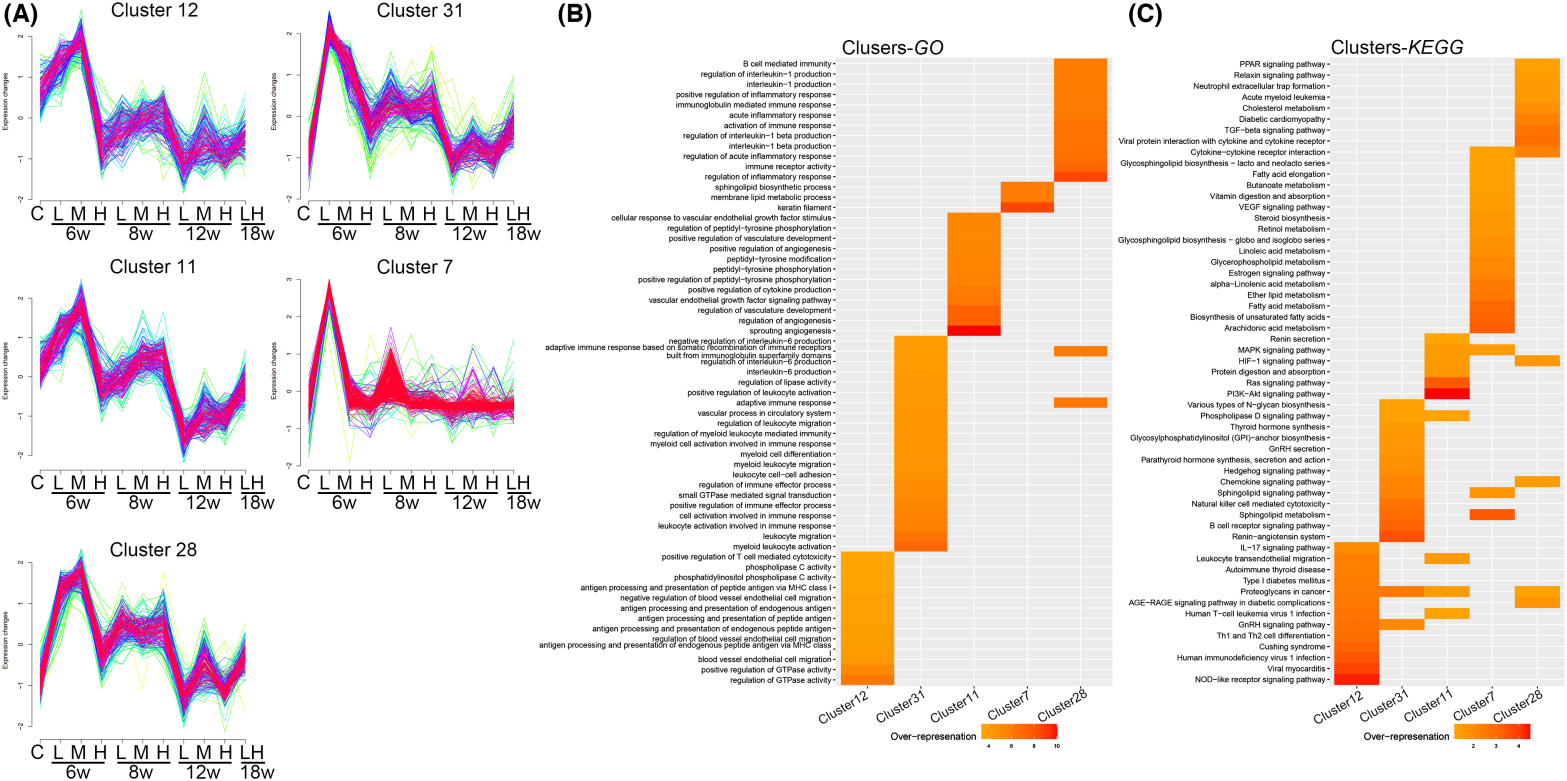
Cluster and enrichment analyses of time‐series genes. (A) The analysis was carried out for Clusters 7/11/12/28/31; (B) Gene Ontology (GO) analysis; (C) Kyoto Encyclopedia of Genes and Genomes (KEGG) analysis.

DEGs were subjected to a time‐series cluster analysis to identify gene clusters with broadly consistent expression trends. The DEGs were divided into 40 clusters (Figure S1). As seen in Figure 2, many genes associated with muscle remodelling showed a downward temporal gradient, so we selected five representative clusters for analysis (Figure 3). In Cluster 12, DEGs were significantly upregulated in the Low and Medium groups at 6 weeks compared to the control group. Although DEGs could be slightly upregulated with increasing exercise intensity at 8 weeks, the overall expression of DEGs was still lower than the control group at 8, 12 and 18 weeks. GO/KEGG analysis revealed that these DEGs were mainly associated with antigen presentation. Cluster 31 DEGs also showed a peak of upregulation at 6 weeks, followed by a temporal decrease in expression. However, the overall expression level was higher than that of the control group, and these DEGs were mainly associated with immune regulation. The expression trend of DEGs in Cluster 11 decreased stepwise over time, while the expression levels increased with increasing exercise intensity between the 8, 12 and 18‐week groups, with the expression levels of DEGs at 12 weeks being significantly lower than those in the control group. Therefore, angiogenesis is not only related to the duration of exercise but also the intensity of exercise and that the two do not appear to have the same effect on the transcriptome levels of angiogenesis. DEGs were transiently upregulated in the Low group at 6 and 8 weeks in Cluster 7. In contrast, the expression levels of DEGs at other time points and groups were similar to controls, and these DEGs were mainly enriched in lipid metabolism and VEGF signalling pathways. The DEGs of Cluster 28 were mainly closely associated with the inflammatory response. They were significantly upregulated at 6 and 8 weeks, while the expression levels were similar to the control group at 12 and 18 weeks.

### 3.3 Gene modules closely connected with time and exercise intensity

Twenty‐one gene modules were divided by WGCNA analysis (Figure S2A). The MEgrey module was strongly positively correlated with time (0.44) and exercise intensity (0.65), whereas the MElightgreen module showed a negative correlation with time (−0.52) and exercise intensity (−0.41). MEpink, MEbrown, MEcyan and MEmagenta were additional modules independently correlated with time (*Q* < 0.05, negative correlation). The modules independently associated with exercise intensity (*Q* < 0.05, positive correlation) included MEblack, MEgrey60, MEsalmon and MEtan.

Modules with significant differences (*Q* < 0.05) were subsequently examined using GO/KEGG analysis. The genes of the MEgrey module were enriched in biological behaviours such as muscle regulation. In contrast, the MElightgreen module genes were primarily enriched in biological processes or molecular functions such as immune regulation (Figure S2B). In addition, the genes in MEpink, MEbrown, MEcyan and MEmagenta modules were mainly enriched in biological processes or signalling pathways such as blood vessel regulation (Figure S2C), suggesting that some of the DEGs that regulated blood vessels showed gradually decreasing expression levels over time. The genes of MEblack, MEgrey60, MEsalmon and MEtan modules were mainly enriched in biological processes or signalling pathways such as muscle remodelling and cell metabolism (Figure S2D), indicating that improvements in these areas were closely linked to increased exercise intensity.

### 3.4 Characteristics of histological chronotropic changes in skeletal muscle remodelling under different exercise loads

**FIGURE 4:**
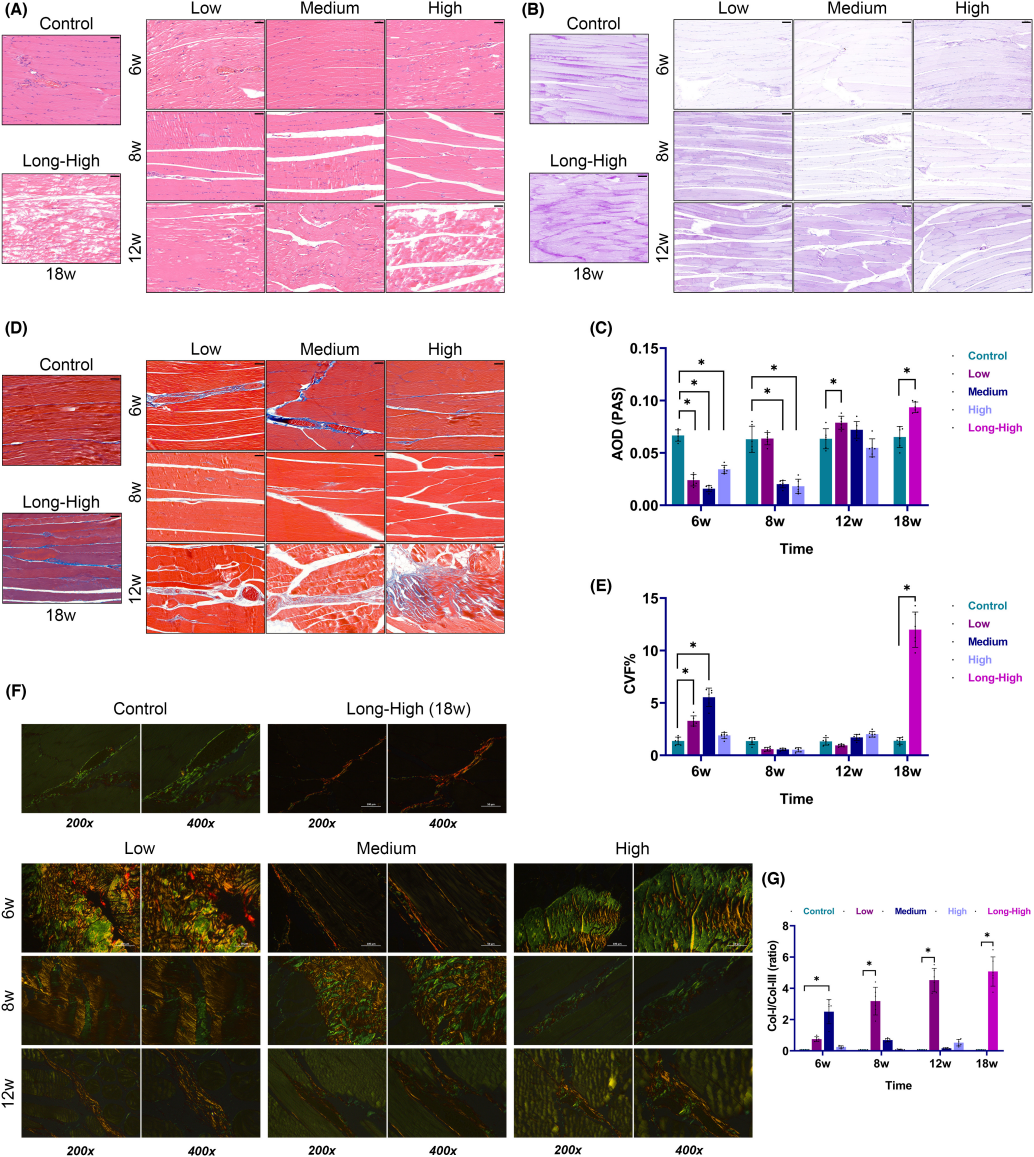
Non‐specific histological features of skeletal muscle. (A) Haematoxylin and eosin staining (scale bar = 50 μm); (B) periodic acid‐schiff (PAS) staining (scale bar = 100 μm); (C) the average optical density (AOD) was used to quantify the glycogen content in PAS staining (*p < 0.05); (D) Masson's trichrome staining (scale bar = 50 μm); (E) statistical diagram of collagen volume fraction (CVF) (CVF% = Collagen area/Total muscle area × 100%, *p < 0.05); (F) sirius red (SR) staining (scale bars = 50 or 100 μm); (G) statistical plot of Type I and III collagen area ratio in SR staining (*p < 0.05).

Haematoxylin and Eosin staining showed partial rupture injury and slightly disordered arrangement of the skeletal muscle fibres of the exercise model rats, compared with the controls. The most significant injury was detected at 12 and 18 weeks while minor signs of muscle fibre injury were seen at 6 weeks (Figure 4A). Muscle glycogen content was considerably lower at the early exercise stage (≤6 weeks), remained low at 8 weeks in the medium and high‐intensity groups, but rebounded in the low‐intensity group, and gradually accumulated in each group at 12 and 18 weeks (Figure 4B,C). Masson's staining significantly distinguishes between collagen fibres and muscle fibres, with the muscle fibres appearing red and the collagen fibres blue. Masson staining revealed increased collagen fibrils in the low and medium‐intensity groups at 6 weeks, while there was no appreciable difference in collagen fibre contents between the above three groups and the control group at 8 and 12 weeks. We also found that the high‐intensity group had significant muscle fibre damage and enhanced intermuscular collagen fibril deposition at 12 weeks. In addition, the long‐term high‐intensity group had significantly more collagen fibrils (Figure 4D,E). SR staining further revealed that muscle collagen fibre type changed with continuous exercise (Figure 4F,G). CoL‐I content transiently increased in the medium‐intensity group at 6 weeks. It kept increasing over time in the low‐intensity group. The CoL‐I content was significantly higher in the long‐term high‐intensity group than in the control group after 18 weeks. However, it appeared unaffected in the high‐intensity group in the early stages of exercise.

**FIGURE 5:**
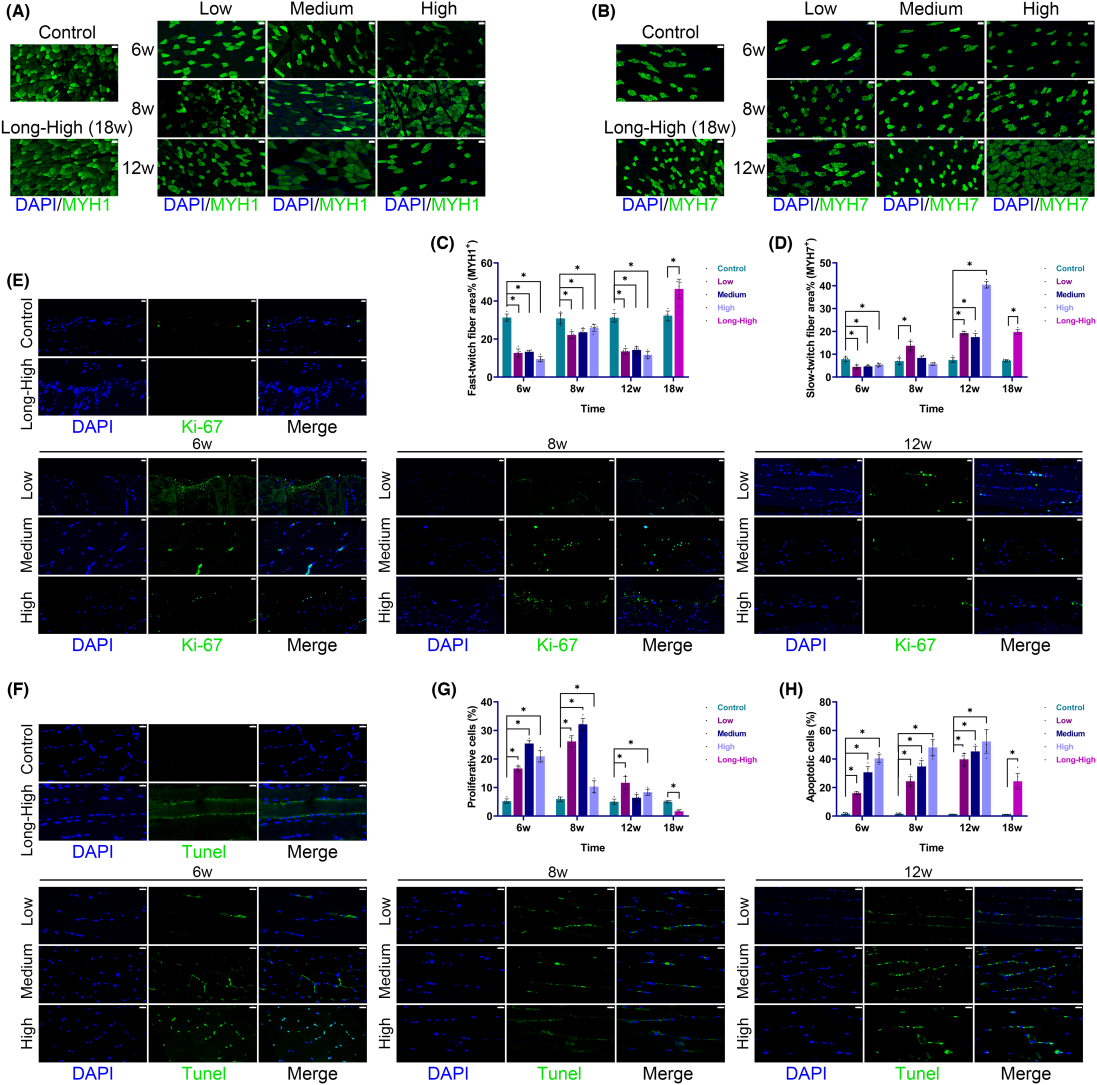
Skeletal muscle fibre types, cell proliferation and apoptosis by immunofluorescence. (A) Fast‐twitch fibres (MYH1^+^) (scale bar = 100 μm); (B) slow‐twitch fibres (MYH7^+^) (scale bar = 100 μm); (C) histogram of fast‐twitch fibre proportion (*p < 0.05); (D) histogram of slow‐twitch fibre proportion (*p < 0.05); (E) proliferating cells (Ki‐67, scale bar = 20 μm); (F) proliferating cells (terminal deoxynucleotidyl transferase dUTP nick end labeling (TUNEL), scale bar = 20 μm); (G) histogram of proliferating cell proportion (*p < 0.05); (H) histogram of apoptotic cell proportion (*p < 0.05).

**FIGURE 6:**
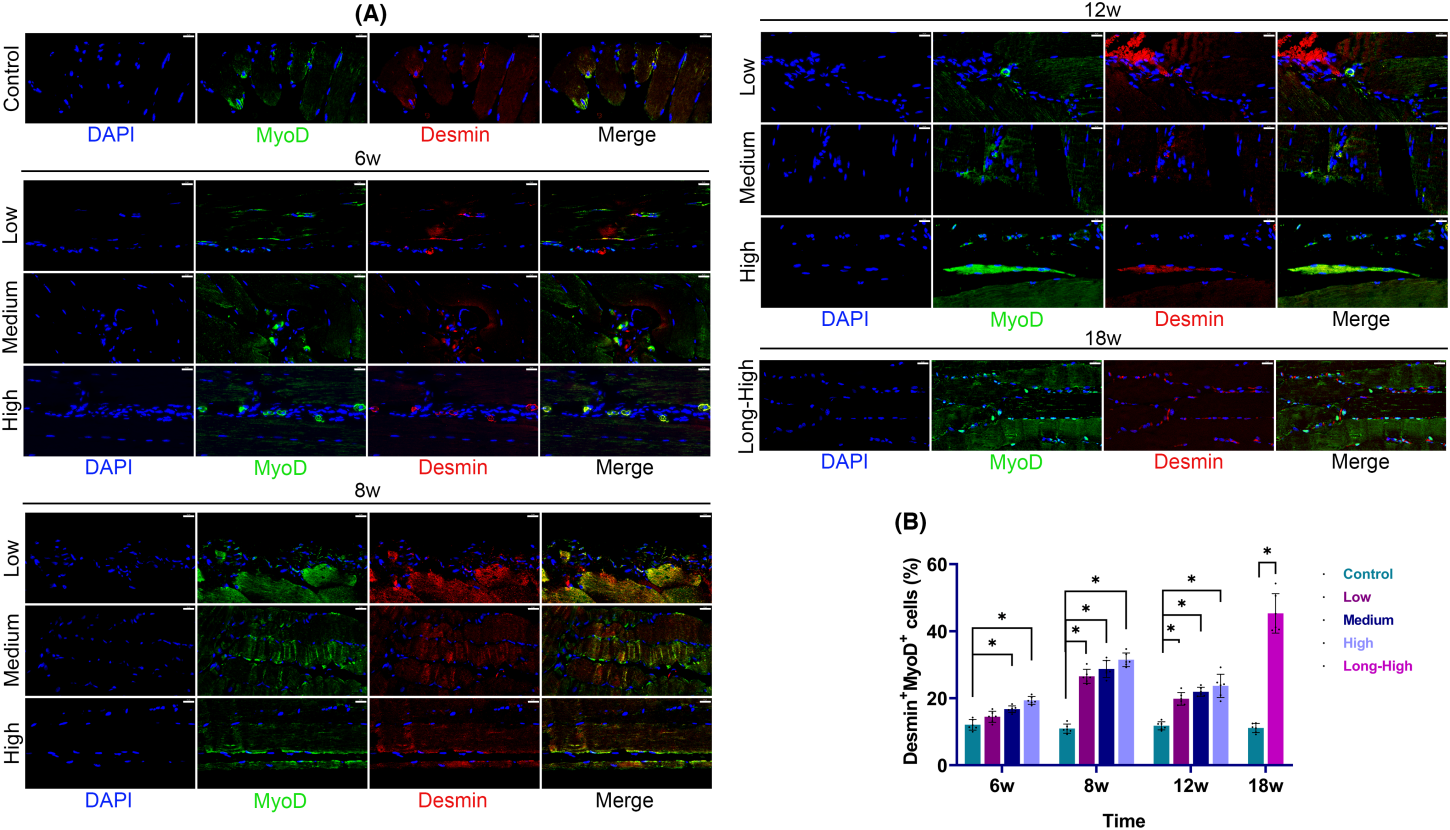
Myoblast proportion and distribution. (A) Immunofluorescence for myoblasts (Desmin^+^MyoD^+^ cells, scale bar = 20 μm); (B) histogram of myoblast proportion (*p < 0.05).

Skeletal muscle fibre types also underwent significant changes. The fast‐twitch fibre (MYH1^+^) proportion was significantly decreased in the three exercise models at 6, 8 and 12 weeks but increased at 18 weeks in the long‐term high‐intensity group. The proportion of slow‐twitch fibres (MYH7^+^) decreased transiently at 6 weeks, whereas in exercise groups (Low/Medium/High/Long‐High) it was significantly higher than that of the control group at 12 and 18 weeks (Figure 5A–D). Additionally, we observed a considerable rise in the proportion of proliferating cells in all groups, followed by a declining trend. However, the proportion of apoptotic cells increased with time and increased exercise intensity (Figure 5E–H). At the same time, we observed that myoblasts (Desmin^+^MyoD^+^ cells), which were mainly located between the epimysium and perimysium, increased dramatically over time and with greater exercise intensity (Figure 6). Therefore, continuous exercise caused significant remodelling of rat skeletal muscle tissue.

### 3.5 Exercise‐induced inflammation and macrophage polarization changed over time

**FIGURE 7:**
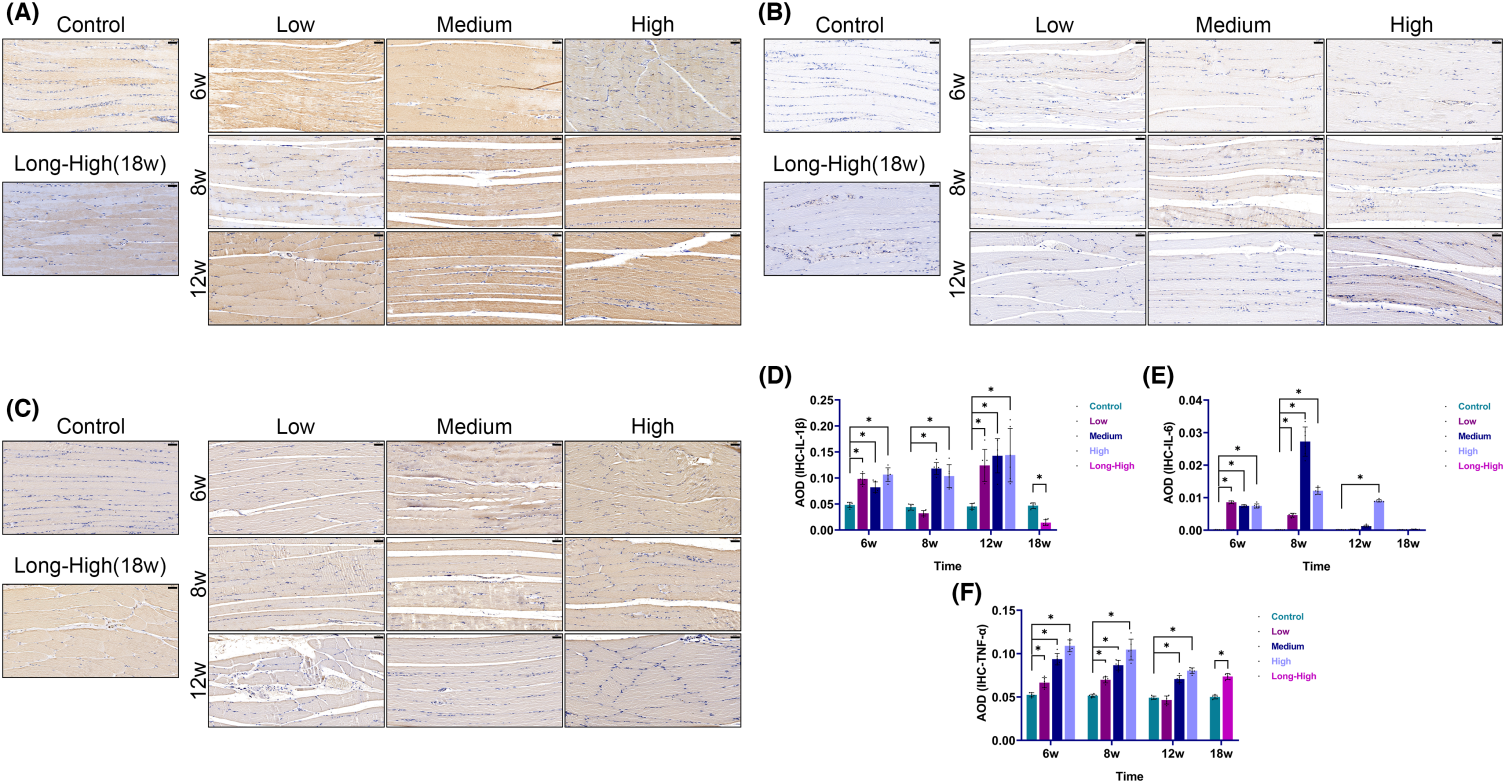
Inflammatory infiltration of skeletal muscle by immunohistochemistry. (A) IL‐1β (scale bar = 50 μm); (B) IL‐6 (scale bar = 50 μm); (C) TNF‐α (scale bar = 50 μm); (D) histogram of IL‐1β average optical density (AOD) (*p < 0.05); (E) histogram of IL‐6 AOD (*p < 0.05); (F) histogram of TNF‐α AOD (*p < 0.05).

**FIGURE 8:**
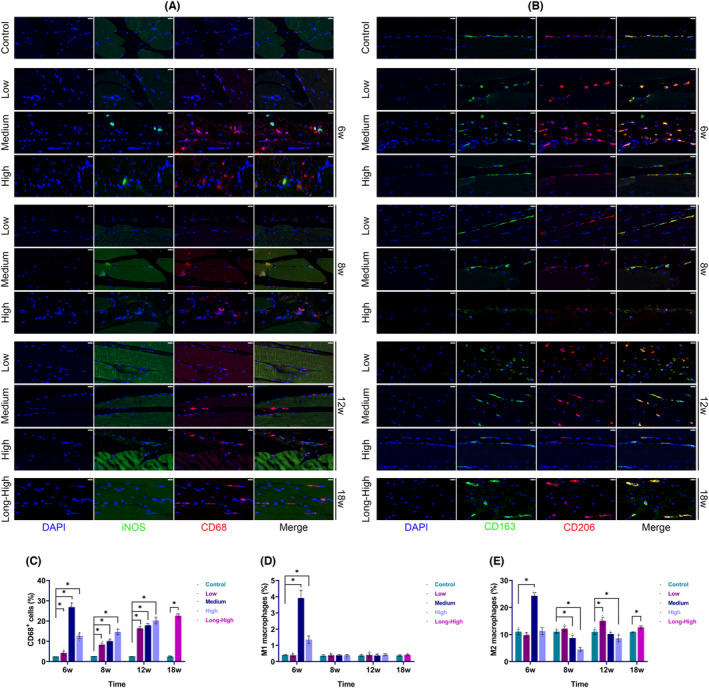
Macrophage infiltration and polarization through immunofluorescence (% = positive cells/total DAPI^+^ nuclei × 100%). (A) M1 macrophages (CD68^+^iNOS^+^ cells, scale bar = 20 μm); (B) M2 macrophages (CD163^+^CD206^+^ cells, scale bar = 20 μm); (C) statistical plot of CD68^+^cell proportion (*p < 0.05); (D) histogram of M1 macrophage proportion (*p < 0.05); (E) histogram of M2 macrophage proportion (*p < 0.05).

Inflammation was one of the essential characteristics of exercise injury. TNF was the predominant cytokine. IL‐1β, IL‐6 and TNF‐α IHC showed significantly higher levels of inflammation in the Low, Medium and High groups, with TNF‐α being the main inflammatory infiltrating factor in the Long‐High group at 18 weeks (Figure 7). Figure 8 shows that CD68^+^ monocytes increased gradually over time and with increasing exercise intensity. M1 macrophages (CD68^+^iNOS^+^ cells) transiently increased in the medium and high‐intensity groups at 6 weeks. The proportion of M1 macrophages in the exercise model did not significantly differ from that in the control group at 8, 12 and 18 weeks. However, M2 macrophages (CD163^+^CD206^+^ cells) transiently increased in the low‐intensity group at 12 weeks and the medium‐intensity group at 6 weeks. In comparison, the ratio of M2 macrophages increased significantly at 18 weeks after dropping at 8 and 12 weeks in the high and long‐term high‐intensity groups. It was evident that there was a vital timing of macrophage polarization during skeletal muscle injury and repair, and the timing change characteristics were closely related to exercise intensity.

### 3.6 Exercise‐induced chronological vascular bed remodelling in the intermuscular and extramuscular myofascial of skeletal muscle

**FIGURE 9:**
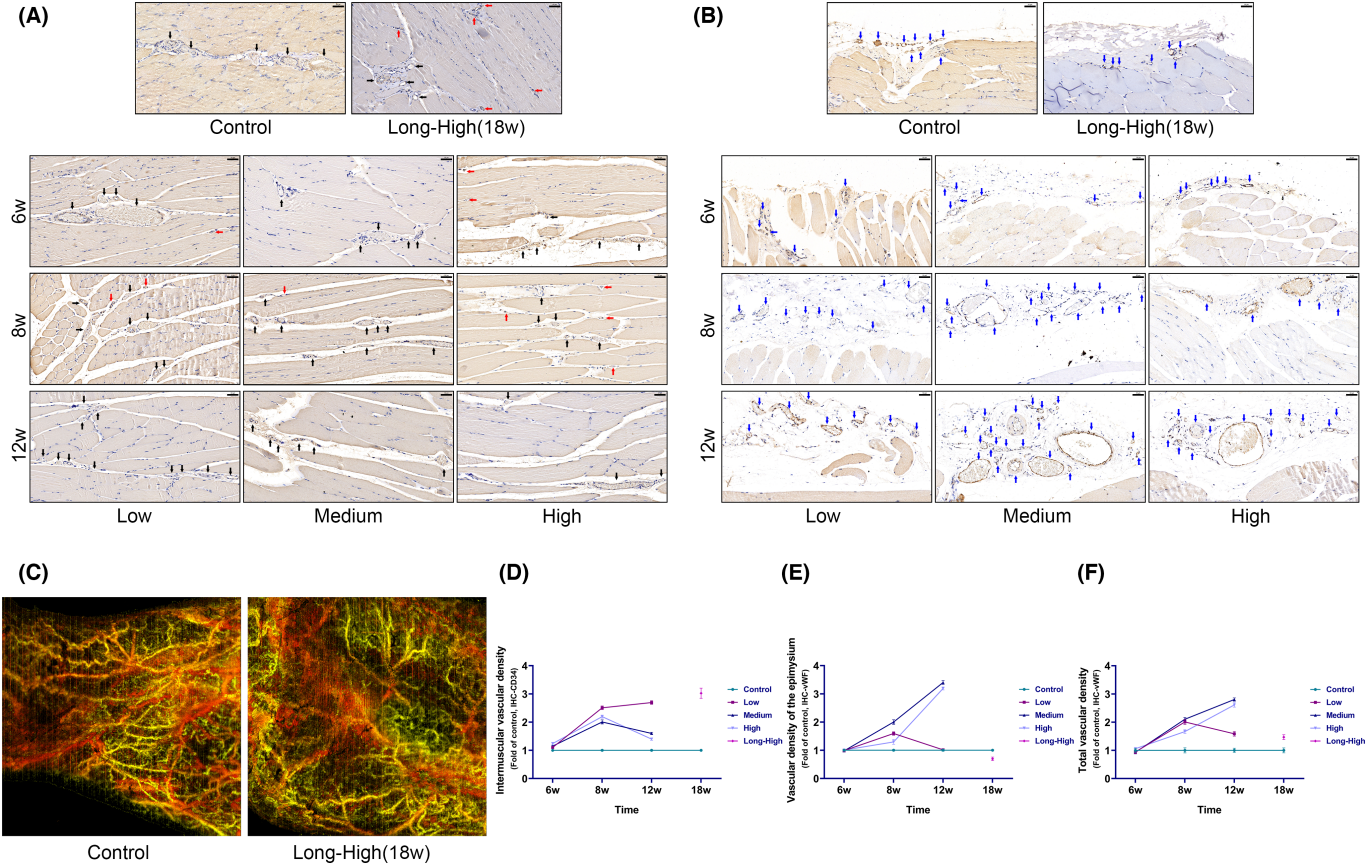
Skeletal muscle vascular remodelling. (A) Immunohistochemistry (IHC) for CD34 showed the proliferation of intermuscular and intramuscular blood vessels (black arrows indicate intermuscular blood vessels, red arrows indicate intramuscular blood vessels, scale bar = 50 μm); (B) IHC for vWF showed epimysial vascular bed remodelling (blue arrows indicate adventitial vessels, scale bar = 50 μm); (C) optical coherence tomography angiography showed fewer vessels with good perfusion function in the long‐term high‐intensity group than in the control group; (D–F) Line plots of the density values of the intermuscular, epicardium and blood vessels.

The blood vessels provide crucial energy needed for tissue injury repair. RNA‐seq showed that some of the common provascular DEGs were highly expressed in the early exercise phase, and their expression levels gradually decreased over time (Figure 3). Interestingly, the intermuscular vessel density of skeletal muscle increased from 6 to 12 weeks in the low‐intensity group, while the epimysial vessels decreased at 12 weeks. In the medium and high‐intensity groups, there was an increase in the intermuscular microvessels from 6 to 12 weeks (a slight decrease was noted at 12 weeks compared with 8 weeks) and a persistent increase in the epimysial vessels. There was a significant increase in intermuscular vessels but an evident decrease in the epimysial vessels in the long‐term high‐intensity group. OCTA imaging showed that, in the long‐term high‐intensity group, vasculature with good perfusion function was fewer at 18 weeks compared to the control group (Figure 9A–E; All statistics are shown in Tables S17 and S18). The overall vascular density of skeletal muscle was higher in the exercise groups than in the control group (Figure 9F, Table 1). Therefore, we are aware that there are spatial differences in the alteration of vascular density following exercise and that the shift in the spatial distribution of blood vessels may be related to muscle remodelling. However, the exact role and mechanisms still need to be clarified.

**TABLE 1:** Simple effect analysis of pairwise comparisons between group and time in all blood vessels.

Total vascular density^a^			Mean difference	*p*	95% confidence interval for difference	
Group	Time (I)	Time (J)	(I–J)		Lower bound	Upper bound
Control	6 w	8 w	−4.441 × 10^−16^	1.000	−0.113	0.113
		12 w	−0.002	1.000	−0.114	0.111
		18 w	−0.002	1.000	−0.114	0.111
	8 w	12 w	−0.002	1.000	−0.114	0.111
		18 w	−0.002	1.000	−0.114	0.111
	12 w	18 w	1.664 × 10^−15^	1.000	−0.113	0.113
Low	6 w	8 w	−1.065*	0.000	−1.167	−0.963
		12 w	−0.643*	0.000	−0.745	−0.542
	8 w	12 w	0.422*	0.000	0.320	0.523
Medium	6 w	8 w	−1.170*	0.000	−1.272	−1.068
		12 w	−1.870*	0.000	−1.972	−1.768
	8 w	12 w	−0.700*	0.000	−0.802	−0.598
High	6 w	8 w	−0.607*	0.000	−0.708	−0.505
		12 w	−1.553*	0.000	−1.655	−1.452
	8 w	12 w	−0.947*	0.000	−1.048	−0.845
**Time**	**Group (I)**	**Group (J)**				
6 w	Control	Low	0.057	0.690	−0.056	0.169
		Medium	0.067	0.514	−0.046	0.179
		High	−0.060	0.632	−0.173	0.053
	Low	Medium	0.010	1.000	−0.103	0.123
		High	−0.117*	0.038	−0.229	−0.004
	Medium	High	−0.127*	0.019	−0.239	−0.014
8 w	Control	Low	−1.008*	0.000	−1.121	−0.896
		Medium	−1.103*	0.000	−1.216	−0.991
		High	−0.667*	0.000	−0.779	−0.554
	Low	Medium	−0.095	0.143	−0.208	0.018
		High	0.342*	0.000	0.229	0.454
	Medium	High	0.437*	0.000	0.324	0.549
12 w	Control	Low	−0.585*	0.000	−0.698	−0.472
		Medium	−1.802*	0.000	−1.914	−1.689
		High	−1.612*	0.000	−1.724	−1.499
	Low	Medium	−1.217*	0.000	−1.329	−1.104
		High	−1.027*	0.000	−1.139	−0.914
	Medium	High	0.190*	0.000	0.077	0.303
18 w	Control	Long‐High	−0.467*	0.000	−0.550	−0.384

^a^ Total vascular density (fold of control) in vWF immunohistochemistry.

* The mean difference is significant at the 0.05 level.

### 3.7 The FHL2/SFRP2 axis is closely related to angiogenesis regulation in skeletal muscle

**FIGURE 10:**
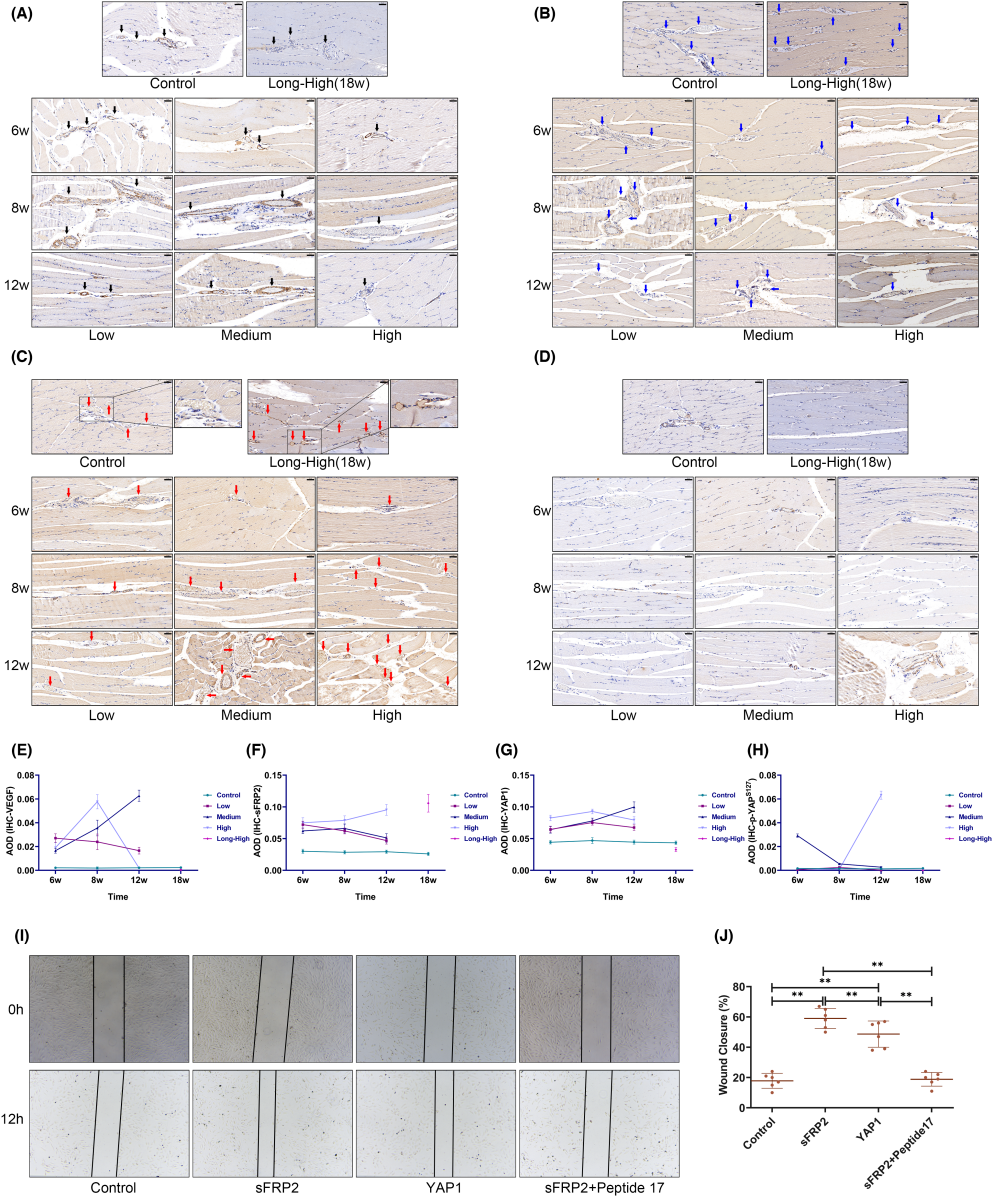
High YAP1 and sFRP2 expressions. (A) Immunohistochemistry (IHC) showed a decreasing trend of VEGFA expression in the low, high and long‐term high‐intensity groups at 12 and 18 weeks (arrows indicate blood vessels, scale bar = 50 μm); (B) IHC demonstrated that high‐intensity exercise (high and long‐term high‐intensity groups) could induce a sustained increase in SFRP2 expression (arrows indicate blood vessels, scale bar = 50 μm); (C) IHC showed that YAP1 expression was elevated in the low, medium and long‐term high‐intensity groups. Although YAP1 expression was decreased in the long‐term high‐intensity group, its expression was elevated around blood vessels (arrows indicate blood vessels, scale bar = 50 μm); (D) IHC for phospho‐YAP1^S127^ revealed a significant increase in the degree of YAP1 phosphorylation in the medium‐intensity group at 6 weeks and the high‐intensity group at 12 weeks, with no significant differences observed in the other groups (arrows indicate blood vessels, scale bar = 50 μm); (E–H) line plots of VEGFA, sFRP2, YAP1 and phospho‐YAP1^S127^ average optical densities; (I) scratch wound healing assay; (J) statistical plot of scratch closure rate (*p < 0.05).

**FIGURE 11:**
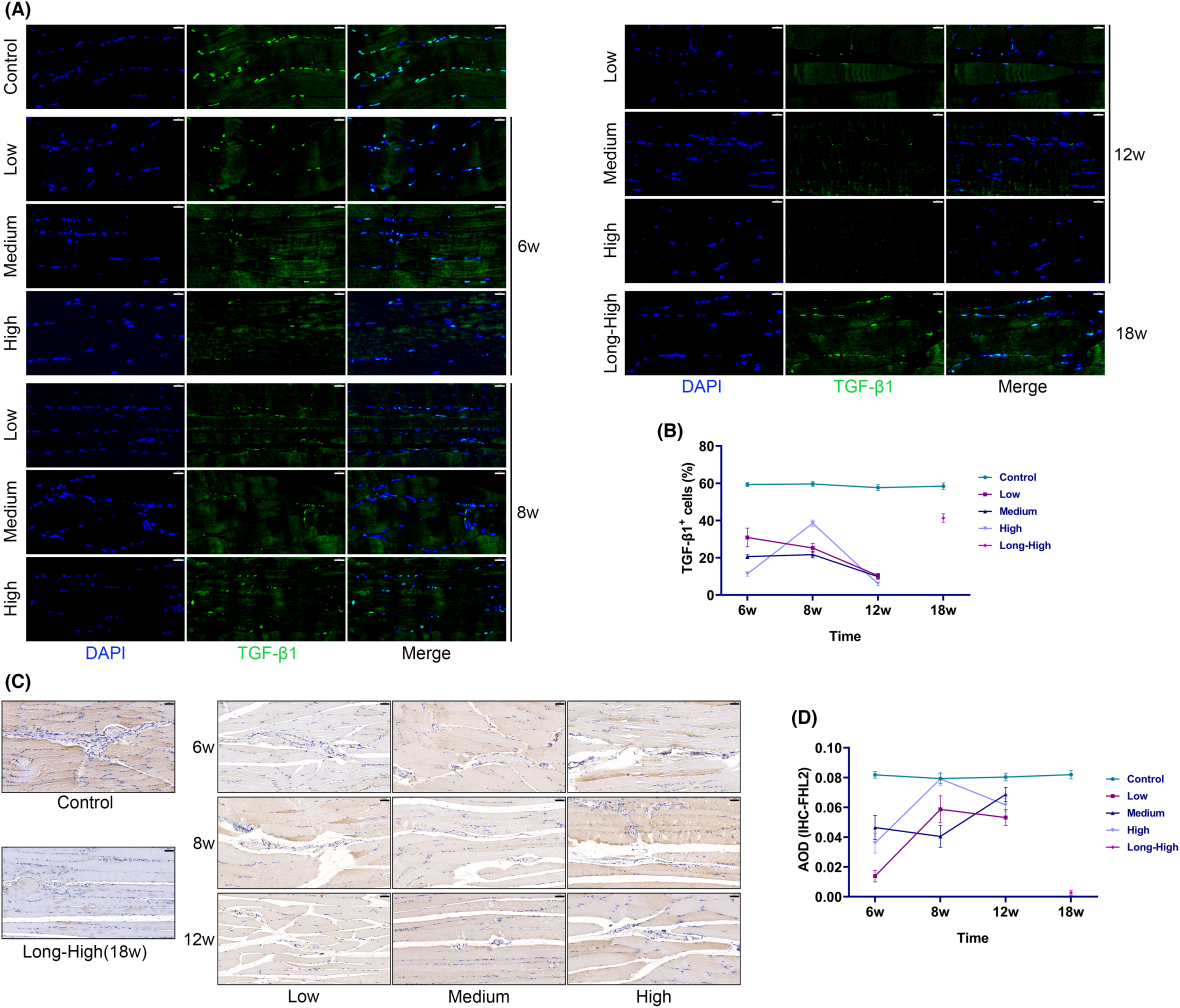
Significant reductions of TGF‐β1 and FHL2. (A) Immunofluorescence for TGF‐β1 (scale bar = 20 μm); (B) line plot of TGF‐β1^+^ cell proportion; (C) immunohistochemistry for FHL2 (scale bar = 50 μm); (D) line plot of FHL2 average optical density.

The volcano plot and correlation heat map show that *TGF‐β1*, *FHL2*, *SFRP2* and *YAP1* gene expressions were significantly temporally different and correlated with common angiogenic genes (Figure S3). IHC data showed that the overall expression of VEGFA, sFRP2 and YAP1 increased at the early exercise stages. However, VEGFA and YAP1 decreased in the high and long‐term high‐intensity groups at 12 and 18 weeks (Figure 10A–H; All statistics in Figure 10 are shown in Tables S19–S22). The high‐intensity group showed persistently elevated sFRP2 and a substantial rise in YAP1 phosphorylation at 12 weeks. Although the overall YAP1 expression decreased in the long‐term high‐intensity group at 18 weeks, it was elevated around the microvessels (Figure 10C). In the scratch assay, sFRP2 and YAP1 significantly promoted endothelial cell migration, while Peptide 17, a YAP1 inhibitor, hindered this effect (Figure 10I,J). Therefore, we propose that sFRP2 is crucial in regulating skeletal muscle angiogenesis during the late phase of EIMI tissue remodelling. We also found that the TGF‐β^+^ cell proportion was significantly reduced (Figure 11A,B), accompanied by a reduction in FHL2, with the lowest value present at 18 weeks (Figure 11C,D).

## 4 DISCUSSION

Skeletal muscle plasticity is highly dynamic, and includes responses to microinjury, physiological or pathological repair and eventual muscle remodelling. EIMI is a widespread concern in athletes. Improvement of physiological muscle receptivity and optimizing training intensity may avoid severe permanent injury.^21^, ^22^ There is a tremendous significance and clinical value in exploring the characteristics of temporal changes in skeletal muscle physiopathology in response to various levels of exercise. In this study, we conducted longitudinal analyses of the transcriptomic, histological and vascular bed remodelling responses of rat skeletal muscle to various exercise paradigms and showed that vascular bed remodelling is a critical pathological feature of skeletal muscle remodelling.

Previous studies had demonstrated that temporary skeletal muscle injury can be induced in rats by horizontal treadmill exercise at a speed of 15–16 m/min for a cumulative period of approximately 90 min or until exhaustion.^23^, ^24^, ^25^ Paola et al. found that, in rats, horizontal treadmill exercise of 15–45 min per day for 2–4 weeks at a speed of 30–45 cm/s can accelerate functional recovery following traumatic muscular injury.^15^, ^26^ Takekura et al. found that running on a treadmill that was either flat or 16° downhill, at a speed of 18 m/min for 5 min (rest interval 2 min and 18 times), can induce skeletal muscle injury and contraction excitation‐coupling failure in rats.^27^ From these data, it is evident that exercise intensity is not the only factor that modulates the repair of skeletal muscle injury. Different exercise durations and patterns are critical for the physiopathological features of skeletal muscle to develop. There is still a lack of systematic, long‐term and multi‐intensity EIMI studies, and the temporal physiopathological characteristics of repair after skeletal muscle exercise injury are unclear. In our study, low‐intensity exercise was able to induce rat skeletal muscle injury or histological changes in microstructure after 6 weeks. We employed an uphill treadmill test, three exercise intensities, and four exercise durations that were selected based on previous studies. We further investigated the physiopathological timing characteristics of EIMI under different exercise modes to identify the critical factors affecting injury repair.

RNA‐seq revealed significant temporal changes in rat skeletal muscle transcriptome profiles in each exercise intensity (Figures 2 and 3). Upregulated DEGs in skeletal muscle dramatically increased between 6 and 8 weeks, declined at 12 weeks and slightly rose again at 18 weeks. Some gene clusters associated with cell catabolism displayed similar trend traits. However, only our high‐intensity exercise group was followed over 18 weeks. We do not know if low‐ and moderate‐intensity exercises present the same chronotropic effects. Although the number of upregulated DEGs increased at 18 weeks, compared with 12 weeks, we found that DEG gene modules and functional characteristics were altered by analysing high and long‐term high‐intensity exercise groups. The temporal expression trend showed that some genes involved in vascularization and muscle remodelling mainly presented features of a dual correlation between time and intensity. For example, the DEGs of Cluster 11 related to vascular remodelling showed a decreasing trend over time; their expression rose with increased exercise intensity.

EIMI transcriptomics may not accurately reflect the ultimate changes in tissue architecture. The injuries may include primary and secondary sarcolemmal disruption, sarcotubular system swelling or disruption, myofibre contractile component disruption, cytoskeletal damage and extracellular myofibre matrix abnormalities.^28^ We saw that muscle fibre tissue was severely damaged at 12 and 18 weeks, and the amount of glycogen content had dramatically decreased. There was a significant change in the proportion of collagen fibres at 12 weeks. It was interesting to see that the glycogen content accumulated at 12 and 18 weeks. High‐intensity exercise required a high metabolic level and fast muscle fibre activity. The continuous training was crucial for slow muscle fibres, and their dependent metabolic pathways were relatively altered.^29^ We found that myoblasts were markedly stimulated, and the muscle fibre type was significantly altered over time in response to the different exercise intensities (Figures 5 and 6). It was evident that tissue remodelling had occurred in rat skeletal muscle. We further showed that the exercise groups had more fast‐ and slow‐twitch fibres than the control group at 18 weeks. However, the impacts of muscle remodelling on subjective feelings, including muscle endurance, explosive power and soreness, remain unclear.

Inflammatory infiltration is an essential pathological feature of tissue injury repair. The characteristics and time‐contingent appropriateness of the response determine whether it has a good or detrimental impact on tissue repair. As an important etiological factor of tissue soreness, persistent inflammation is one of the factors involved in remodelling and impairing the tissue microenvironment.^4^, ^17^ However, inflammatory signals are also critical in initiating tissue repair.^30^ The homeostatic control of pro‐and anti‐inflammatory mediators is essential for the orderly, timely and controlled regulation of inflammation. Using RNA‐seq, we showed that mRNA levels of common inflammatory factors were considerably raised at 6 weeks and then reduced. However, IHC revealed that the total levels of IL‐1β, IL‐6 and TNF‐α rose with increased training load, especially for TNF‐α. Whether this ongoing inflammation exacerbated the injury or promoted self‐repair was not resolved by this study. Nevertheless, research has revealed that persistent muscle inflammation is one of the fundamental causes of exercise‐induced muscle soreness,^31^ and that anti‐inflammatory interventions are beneficial for repair after muscle injury.^32^ Macrophages play a vital role in regulating inflammation. Chazaud et al.^33^ suggested that an appropriate balance of macrophage phenotypes is important for establishing inflammatory microenvironment homeostasis that is favourable for skeletal muscle regeneration. We found in our study that in the low‐intensity exercise group, there were greater numbers of M2 than M1 macrophages. M2 macrophages decreased in the medium and high‐intensity groups compared to the control group, which was consistent with the secretion of pro‐inflammatory factors in these two groups. M2 macrophages unexpectedly increased in the long‐term high‐intensity group at 18 weeks. Macrophages have been found to affect tissue repair positively.^34^, ^35^ Nevertheless, we demonstrated that significant tissue remodelling had already occurred in skeletal muscle at 18 weeks, and it remains to be determined whether an M2 increase constituted a defence mechanism to improve tissue repair in the body.

There is a significant increase in the delivery and absorption of oxygen and nutrients during exercise to meet the metabolic demands of contracting muscles. Increased muscle capillarization following exercise is a hallmark adaptation to exercise.^1^ We found that the expression of angiogenesis‐related gene clusters increased markedly during the beginning of training and then trended downward (Figure 3). However, increased exercise intensity promoted the upregulation of these DEGs. Using IHC we showed that exercise enhanced vascularization in muscle. Even though the vascular density increased at 18 weeks in the long‐term high‐intensity group, an OCTA scan showed a modest decline in perfusion (Figure 9). The increased blood vessels provide a safeguard for energy supply. Nevertheless, normal vascular perfusion is required.^36^ Studies have shown that physiological angiogenesis is necessary for skeletal muscle regeneration and repair.^37^ However, it is equally important to avoid abnormal pathological vascular proliferation.^38^ High permeability and poor perfusion are traits of pathological blood arteries, which impair the body's ability to enhance the movement of oxygen free radicals or inflammatory cells, altering the balance of the tissue microenvironment, and impairing tissue repair. Although blood vessels were found to be hyperplastic, IHC showed a significant decrease in VEGFA expression in the high and long‐term high‐intensity groups at 12 and 18 weeks. In studies on melanoma and chronic myocardial ischemia, elevated SFRP2 was found to be a crucial component of pathological vascular proliferation; VEGF antagonism by advanced pathological vascular proliferation did not limit angiogenesis.^39^, ^40^ sFRP2 and perivascular YAP1 were consistently overexpressed in our high‐ and long‐term high‐intensity groups. Using RNA‐seq, we showed significant temporal differences in *TGF‐β1*, *FHL2*, *sFRP2* and *YAP1* expressions. YAP1 is a vital regulator of the Hippo signalling pathway and plays an essential role in tissue growth and development and the control of skeletal muscle fibre size.^41^ Therefore, we speculated that skeletal muscle remodelling and angiogenesis were strongly associated with the decline in TGF‐β1 and FHL2 and increased sFRP2 and YAP1 expression. The underlying regulatory mechanisms remain to be investigated.

There were some limitations. First, although this work examined the pathological features of rat EIMI such as the transcriptomic, histological, immunomodulatory and vascular remodelling, it did not profoundly explore the exact regulatory mechanisms of FHL2/sFRP2 and Hippo pathways. We seek to investigate the molecular mechanisms of angiogenesis during EIMI in future work. Second, we examined the effects of low‐ and moderate‐intensity exercise on rat skeletal muscle and only identified the pathological features of high‐intensity training at 18 weeks. Our pre‐experiments showed that the exercise intensity in the High group (25 m/min, 15° uphill, 1.5 h/d, 6 d/w) was close to the maximum tolerated exercise intensity, and we wanted to explore whether prolonging exercise at this intensity would alter the pathological features of EIMI, so there was only one exercise intensity at 18 weeks. We do not have more funding to add more exercise conditions. More exploration of EIMI pathology at more exercise intensities will be one of our future research directions. Haematoxylin and Eosin staining has shown significant structural damage to the muscle. However, we do not deny that there is physiological remodelling of some of the muscles. This study did not construct a permanent model of severe dysfunctional muscle damage (complete layer rupture), so presenting the histological features of the more severe EIMI in the clinic is challenging. Future research should expand the duration and intensities of experimental exercise regimens.

## 5 CONCLUSION

This study examines the pathological characteristics of rat skeletal muscle injury and repair in response to various exercise intensities. We identify the temporally controlled processes underlying EIMI using transcriptomics, histology and angiogenesis assessments, which have important implications for exploring intervention time windows or different time point intervention options. We find significant differences in the spatial distribution of angiogenesis during muscle injury‐remodelling, which be helpful for the future achievement of spatially targeted treatments for EIMI.

## AUTHOR CONTRIBUTIONS

**Qihang Su:** Conceptualization (lead); formal analysis (lead); methodology (lead); project administration (lead); resources (lead); visualization (lead); writing – original draft (lead). **Jie Li:** Conceptualization (equal); formal analysis (equal); methodology (equal); project administration (equal); resources (equal); visualization (equal); writing – original draft (equal). **Jingbiao Huang:** Conceptualization (equal); formal analysis (equal); methodology (equal); project administration (equal); resources (equal); visualization (equal); writing – original draft (equal). **Qiuchen Cai:** Investigation (equal); project administration (equal); resources (equal). **Chao Xue:** Investigation (equal); methodology (equal); resources (equal). **Chenglong Huang:** Formal analysis (equal); investigation (equal); resources (equal). **Liyang Chen:** Investigation (equal); resources (equal). **Jun Li:** Investigation (equal); resources (equal). **Dandan Li:** Funding acquisition (equal); supervision (equal); writing – review and editing (equal). **Hengan Ge:** Funding acquisition (equal); supervision (equal); writing – review and editing (equal). **Biao Cheng:** Funding acquisition (equal); supervision (equal); writing – review and editing (equal).

## FUNDING INFORMATION

The study was funded by the National Natural Science Foundation of China (81972095, 82272529, 81901754), the Innovation Action Plan of Science and Technology Commission of Shanghai Municipality of China (20S31901400, 19441901700, 19441901701, 19441901702), the Project of Shanghai Hospital Development Center (SHDC12022101), the Project of Shanghai Health Commission (20194Y0385) and the Development Project of Shanghai Tongji Hospital (GJPY2221, GJPY2223, GJPY2230).

## CONFLICT OF INTEREST STATEMENT

The authors declared no potential conflicts of interest with respect to the research, authorship and/or publication of this article.

## Supporting information


Data S1:

Figure S1.

Figure S2.

Figure S3.

Table S1.

Table S2.

Table S3.
Table S4.
Table S5.
Table S6.
Table S7.
Table S8.
Table S9.
Table S10.
Table S11.
Table S12.
Table S13.
Table S14.
Table S15.
Table S16.
Table S17.
Table S18.
Table S19.
Table S20.
Table S21.
Table S22.
Table S23.
Table S24.

## Data Availability

All data generated or analysed during this study are included in this published article and available from the corresponding author upon reasonable request.
