# Supplementary material for: Histological characteristics of exercise‐induced skeletal muscle remodelling
Source: J Cell Mol Med. 2023 Jul 30;27(21):3217–34. doi: 10.1111/jcmm.17879 (PMC10623533; doi:10.1111/jcmm.17879)
Supplement: Supplementary file 1 — Data S1: [file JCMM-27-3217-s006.docx]

**Supplementary Methods**

**Histological staining**

HE was performed using a standard staining set (Wuhan Servicebio Technology Co., Ltd.). The sections were first stained in a hematoxylin solution for three to five minutes, then rinsed with water. Then, the sections were processed with Hematoxylin Differentiation solution, rinsed with water, treated with Hematoxylin Scott Tap Bluing reagent, then rinsed with running water. Subsequently, the sections were dehydrated with 85% and 95% graded ethanol and stained with eosin solution for 5 minutes. Finally, the sections were dehydrated, mounted, and viewed under a microscope.

PAS was performed using a standard staining set (Wuhan Servicebio Technology Co., Ltd.). The sections were stained with PAS staining solution B for 10 to 15 minutes, then rinsed twice with distilled water. Next, the sections were immersed in PAS staining solution A for 25 to 30 minutes, shielded from light, and rinsed in running water for 5 minutes. Then the sections were mounted in PAS stain solution C for 30 seconds, then rinsed with water. Next, the slices were treated with Hydrochloric acid solution and Ammonia. Each step required rinsing with water. Finally, the sections were dehydrated, mounted, and examined under a microscope. The nucleus appeared light blue, while the glycogen in the stained tissue was purple. The average optical density (AOD) was measured to quantify glycogen storage by Image-Pro Plus 6.0 software.

Masson was performed using a standard staining set (Wuhan Servicebio Technology Co., Ltd.). First, the sections were soaked in Masson solution A overnight, then rinsed with water. Then the sections were immersed in an equal mixture of Masson B and C solutions for one minute, washed with water, differentiated with 1% hydrochloric acid alcohol, and rewashed with water. After 6 minutes in the Masson D solution, the sections were washed with water. The sections were then directly stained with Masson F solution for 2 to 30 seconds after being submerged in Masson E solution for one minute. Finally, the sections were rinsed, differentiated with 1% glacial acetic acid, dehydrated, mounted, and examined microscopically. Collagen fibers appeared blue, and muscle fibers showed red in the stained tissue. Collagen and muscle fiber areas were measured using Image-Pro Plus 6.0 software to evaluate collagen volume fraction (CVF) (CVF%=Collagen area/Total muscle area×100%) ^19^.

SR was performed by using a standard staining set (Wuhan Servicebio Technology Co., Ltd.). The sections were stained in SR staining solution for 8 minutes, dehydrated, mounted, and viewed by polarized light microscopy (Nikon Eclipse Ci, NIKON digital sight DS-FI2). Collagen type I (Col-I) showed orange or bright red thick fibers in the stained tissue. Collagen type III (Col-III) appeared with thin green fibers. Col-I and COL III areas and their ratio (Col-I/Col-III) were measured using Image-Pro Plus 6.0 software.

**Immunofluorescence (IF)**

MYH1 (1:1000), MYH7 (1:1000), Ki-67 (1:1000), and TGF-β1 (1:800) were detected by IF staining. Primary antibodies included GB112130, GB112131, GB111499, and GB11179 (Wuhan Servicebio Technology Co., Ltd.). MyoD (primary antibody A0671, 1:50) (ABclonal Co. Ltd., Shanghai, China)+Desmin (1:1000), iNOS (1:500)+CD68 (1:200), CD163 (1:1000)+CD206 (1:800) (primary antibodies GB12075, GB11119, GB113109, GB113751, and GB113497) (Wuhan Servicebio Technology Co., Ltd.) were assessed using double IF staining. The prepared sections were subjected to antigen retrieval (EDTA antigen retrieval buffer, PH8.0) and blocked with 3% BSA (Wuhan Servicebio Technology Co., Ltd.) for the double IF labeling. The first primary antibodies (MyoD, iNOS, and CD163), the corresponding secondary antibodies, and the TSA working solution (G1226, Wuhan Servicebio Technology Co., Ltd.) were added after incubation at room temperature. The primary and secondary antibodies bound to the tissue were removed by microwave after incubation and decolorization. The second primary antibodies (Desmin, CD68, and CD206) were added along with the corresponding secondary antibodies. Finally, the sections were autofluorescence quenched, mounted, and images were acquired using a fluorescence microscope. After adding the primary and secondary antibodies for MYH1, MYH7, Ki-67, and TGF-β1, DAPI counterstaining and subsequent steps could be performed. Finally, Image-Pro Plus 6.0 software was used to measure the fluorescence levels to indicate target expression in positive cells.

**Immunohistochemistry (IHC)**

Targets included IL-1β (1:800), IL-6 (1:800), TNF-α (1:500), CD34 (1:500), vWF (1:1000), VEGF (1:100), sFRP2 (1:100), YAP1 (1:100), Phospho-YAP1^S127^ (1:100), and FHL2 (1:200) (Primary antibodies, GB11113, GB11117, GB11188, GB111693, GB11020, Wuhan Servicebio Technology, Co. Ltd.; A12303, A5383, A1002, AP0489, A1907, ABclonal Co.Ltd., Shanghai, China) ^36^. Sections were first subjected to antigen retrieval utilizing citric acid antigen retrieval buffer (pH 6.0). Endogenous peroxidase was blocked using 3% aqueous hydroxide. The sections were blocked with 3% BSA. Diluted primary antibodies were added for an overnight incubation, followed by secondary antibodies (HRP labeled) with the related species of the primary antibodies. DAB chromogenic solution was added dropwise after washing. The color development was terminated by rinsing the sections with water. Finally, hematoxylin was used to counterstain the nuclei. The sections were dehydrated, mounted, and imaged using a microscope. Vascular density was quantified by IHC results for CD34 and vWF. The expression levels of each indicator were then quantified using Image Pro Plus 6.0 software.

**Terminal deoxynucleotidyl transferase dUTP nick end labeling (TUNEL) assay**

TUNEL assay was performed on sections utilizing conventional methods to quantify the apoptotic cell proportion ^37^. First, the proteinase K working solution (the ratio of proteinase K stock and PBS was 1:9) (G1234, Wuhan Servicebio Technology Co., Ltd.) was dropped into the sections to cover the tissues and placed in an incubator at 37°C for 22 minutes. Then the sections were shaken and rinsed in PBS (pH 7.4) using a decolorizing shaker three times for 5 minutes each. After the sections were slightly shaken dry, membrane-breaking working solution (0.1% triton, G1204, Wuhan Servicebio Technology Co., Ltd.) was added dropwise to cover the tissue and incubated at room temperature for 20 minutes. Next, the sections were placed in PBS and washed on a decolorizing shaker three times for 5 minutes each. The tissues were covered with buffer incubation at room temperature for 10 minutes after the sections were slightly shaken dry. The appropriate amounts of TDT enzyme, dUTP, and buffer in the TUNEL kit (G1501, Wuhan Servicebio Technology Co., Ltd.) were mixed at 1: 5: 50 and added to cover the tissue. The sections were placed flat inside a humid box and incubated at 37°C in an incubator for two hours. A small amount of water was added to maintain humidity. Next, the sections were washed three times with PBS for 5 minutes each time, followed by drops of DAPI staining solution (G1012, Wuhan Servicebio Technology Co., Ltd.), and incubated in the dark for 10 minutes at room temperature. Finally, the sections were mounted, and images were observed and captured under a fluorescence microscope (Nikon Eclipse C1, Japan).
