# Supplementary figures and images for: Histological characteristics of exercise‐induced skeletal muscle remodelling
Source: J Cell Mol Med. 2023 Jul 30;27(21):3217–34. doi: 10.1111/jcmm.17879 (PMC10623533; doi:10.1111/jcmm.17879)

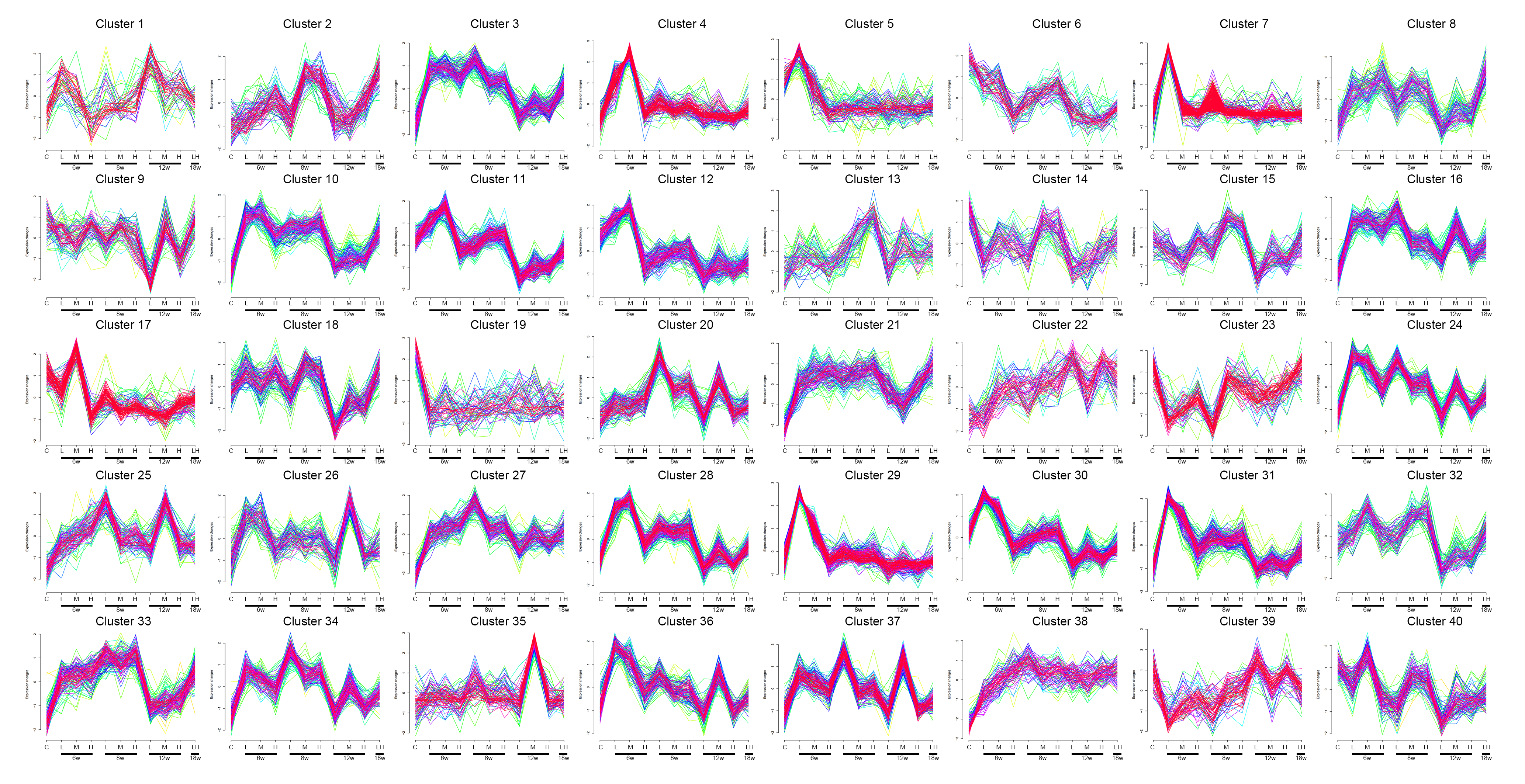

Supplement: Supplementary file 2 — Figure S1. [file JCMM-27-3217-s005.tif]

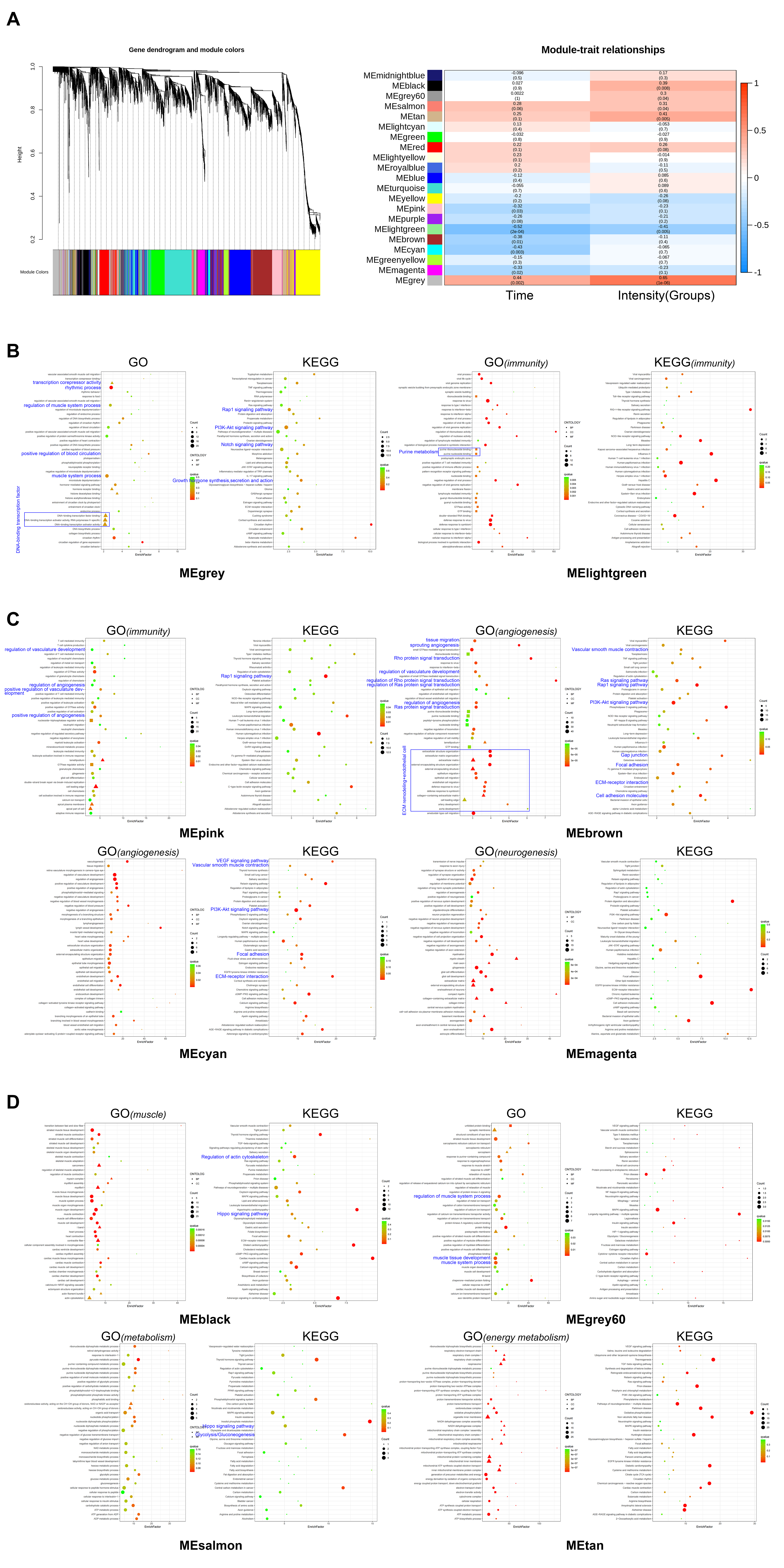

Supplement: Supplementary file 3 — Figure S2. [file JCMM-27-3217-s003.tif]

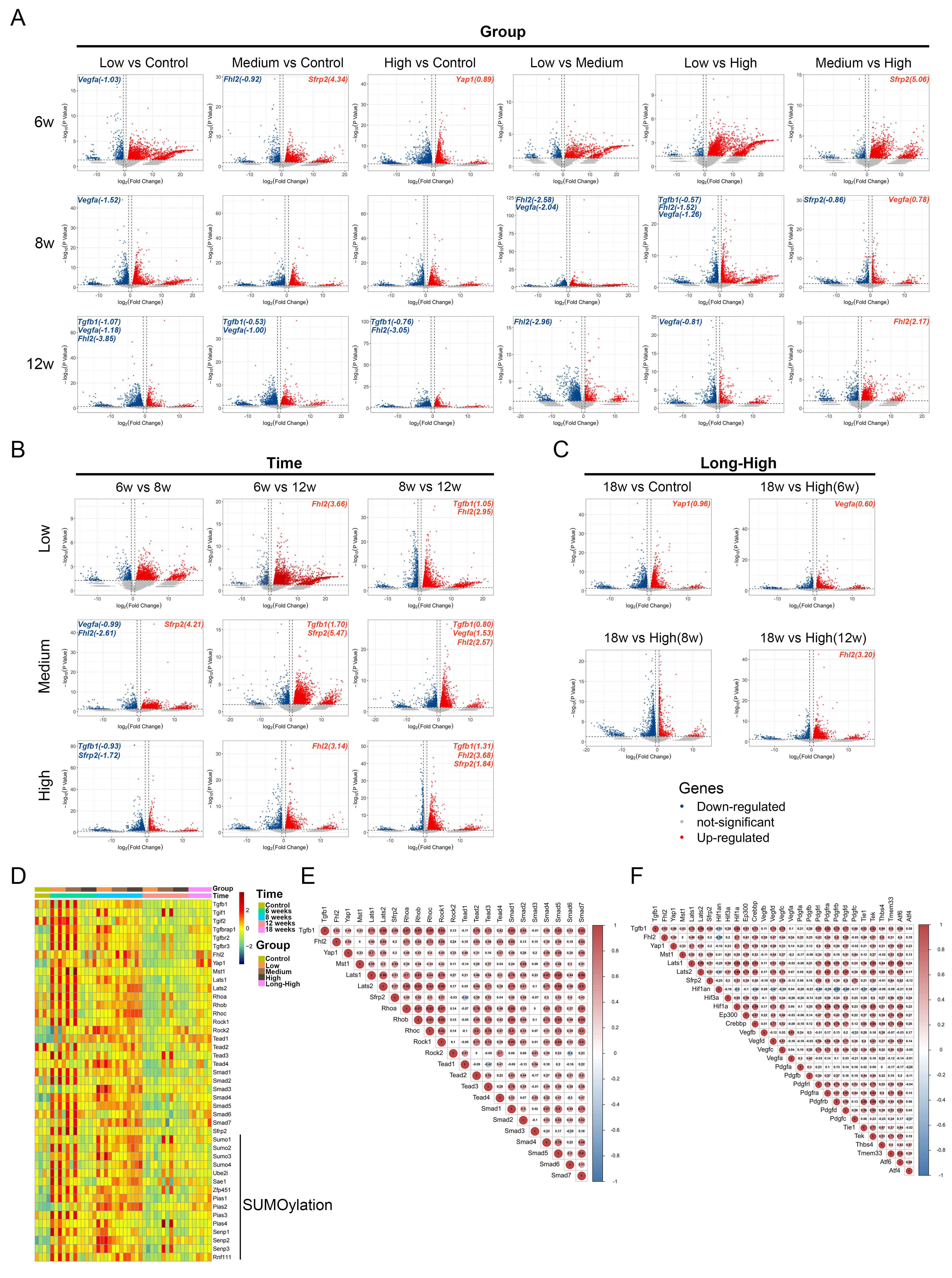

Supplement: Supplementary file 4 — Figure S3. [file JCMM-27-3217-s001.tif]
