# Supplementary material for: Histological characteristics of exercise‐induced skeletal muscle remodelling
Source: J Cell Mol Med. 2023 Jul 30;27(21):3217–34. doi: 10.1111/jcmm.17879 (PMC10623533; doi:10.1111/jcmm.17879)
Supplement: Supplementary file 5 — Table S1. [file JCMM-27-3217-s004.docx]

| **Table S1. Analysis of variance of factorial design** | | | |
| --- | --- | --- | --- |
| Variables# | | *F* | *P* |
| PAS | Time | 89.651 | 0.000 |
|  | Group | 65.220 | 0.000 |
|  | Time*Group | 36.902 | 0.000 |
| Masson | Time | 63.846 | 0.000 |
|  | Group | 266.276 | 0.000 |
|  | Time*Group | 25.824 | 0.000 |
| SR | Time | 3.748 | 0.015 |
|  | Group | 191.209 | 0.000 |
|  | Time*Group | 48.372 | 0.000 |
| IF-MYH1 | Time | 71.236 | 0.000 |
|  | Group | 166.093 | 0.000 |
|  | Time*Group | 16.016 | 0.000 |
| IF-MYH7 | Time | 874.705 | 0.000 |
|  | Group | 277.616 | 0.000 |
|  | Time*Group | 373.996 | 0.000 |
| IF-Ki-67 | Time | 260.645 | 0.000 |
|  | Group | 316.426 | 0.000 |
|  | Time*Group | 137.148 | 0.000 |
| TUNEL | Time | 40.545 | 0.000 |
|  | Group | 356.345 | 0.000 |
|  | Time*Group | 10.413 | 0.000 |
| IF-MyoD+Desmin | Time | 54.402 | 0.000 |
|  | Group | 233.384 | 0.000 |
|  | Time*Group | 11.704 | 0.000 |
| IHC-IL-1β | Time | 16.365 | 0.000 |
|  | Group | 37.968 | 0.000 |
|  | Time*Group | 9.460 | 0.000 |
| IHC-IL-6 | Time | 175.701 | 0.000 |
|  | Group | 232.303 | 0.000 |
|  | Time*Group | 168.466 | 0.000 |
| IHC-TNF-α | Time | 60.270 | 0.000 |
|  | Group | 235.617 | 0.000 |
|  | Time*Group | 8.668 | 0.000 |
| IF-CD68 | Time | 107.813 | 0.000 |
|  | Group | 913.307 | 0.000 |
|  | Time*Group | 178.849 | 0.000 |
| IF-CD68+iNOS | Time | 278.101 | 0.000 |
|  | Group | 168.126 | 0.000 |
|  | Time*Group | 221.896 | 0.000 |
| IF-CD163+CD206 | Time | 115.697 | 0.000 |
|  | Group | 109.747 | 0.000 |
|  | Time*Group | 155.669 | 0.000 |
| IHC-Intermuscular vascular density | Time | 663.840 | 0.000 |
|  | Group | 1427.346 | 0.000 |
|  | Time*Group | 258.998 | 0.000 |
| IHC-Vascular density of the epimysium | Time | 2050.546 | 0.000 |
|  | Group | 1448.035 | 0.000 |
|  | Time*Group | 1243.837 | 0.000 |
| IHC-Total vascular density | Time | 839.689 | 0.000 |
|  | Group | 476.678 | 0.000 |
|  | Time*Group | 271.594 | 0.000 |
| IHC-VEGF | Time | 55.430 | 0.000 |
|  | Group | 221.656 | 0.000 |
|  | Time*Group | 172.716 | 0.000 |
| IHC-sFRP2 | Time | 2.105 | 0.107 |
|  | Group | 261.766 | 0.000 |
|  | Time*Group | 14.617 | 0.000 |
| IHC-YAP1 | Time | 21.851 | 0.000 |
|  | Group | 212.134 | 0.000 |
|  | Time*Group | 28.868 | 0.000 |
| IHC-p-YAPS127 | Time | 783.592 | 0.000 |
|  | Group | 1253.937 | 0.000 |
|  | Time*Group | 2457.531 | 0.000 |
| IF-TGF-β1 | Time | 236.910 | 0.000 |
|  | Group | 1367.556 | 0.000 |
|  | Time*Group | 109.997 | 0.000 |
| IHC-FHL2 | Time | 84.392 | 0.000 |
|  | Group | 307.743 | 0.000 |
|  | Time*Group | 53.177 | 0.000 |
| #Preliminary ANOVA results for each experimental statistics. | | | |
| *Interaction between Group and Time; P<0.05 indicates a significant interaction effect between group and time. | | | |
|  |  |  |  |
|  |  |  |  |
| PAS: Periodic Acid-Schiff staining; Masson: Masson's trichrome staining; SR: Sirius-Red histological staining; IF: Immunofluorescence; IHC: Immunohistochemistry | | | |
