# Supplementary material for: Histological characteristics of exercise‐induced skeletal muscle remodelling
Source: J Cell Mol Med. 2023 Jul 30;27(21):3217–34. doi: 10.1111/jcmm.17879 (PMC10623533; doi:10.1111/jcmm.17879)
Supplement: Supplementary file 6 — Table S2. [file JCMM-27-3217-s002.docx]

| Table S2. Test of Normality and Homogeneity of Variances | | | |
| --- | --- | --- | --- |
|  |  | Statistic | *P* |
| Shapiro-Wilk Test | Control | 0.976 | 0.931 |
|  | sFRP2 | 0.955 | 0.784 |
|  | YAP1 | 0.839 | 0.127 |
|  | sFRP2+Peptide17 | 0.944 | 0.688 |
| Levene's Test |  | 2.270 | 0.112 |
