# Supplementary material for: Histological characteristics of exercise‐induced skeletal muscle remodelling
Source: J Cell Mol Med. 2023 Jul 30;27(21):3217–34. doi: 10.1111/jcmm.17879 (PMC10623533; doi:10.1111/jcmm.17879)
Supplement: Supplementary file 7 — Table S3. Table S4. Table S5. Table S6. Table S7. Table S8. Table S9. Table S10. Table S11. Table S12. Table S13. Table S14. Table S15. Table S16. Table S17. Table S18. Table S19. Table S20. Table S21. Table S22. Table S23. Table S24. [file JCMM-27-3217-s007.docx]

| **Table S3. Simple effect analysis of pairwise comparisons between group and time in Masson's trichrome staining** | | | | | | |
| --- | --- | --- | --- | --- | --- | --- |
| CVF%# | | | **Mean Difference** | ***P*** | **95% Confidence Interval for Difference** | |
| ***Group*** | ***Time (I)*** | ***Time (J)*** | (I－J) |  | **Lower Bound** | **Upper Bound** |
| Control | 6w | 8w | 0.010 | 1.000 | -0.892 | 0.912 |
|  |  | 12w | 0.042 | 1.000 | -0.861 | 0.944 |
|  |  | 18w | -0.013 | 1.000 | -0.916 | 0.889 |
|  | 8w | 12w | 0.032 | 1.000 | -0.871 | 0.934 |
|  |  | 18w | -0.023 | 1.000 | -0.926 | 0.879 |
|  | 12w | 18w | -0.055 | 1.000 | -0.957 | 0.847 |
| Low | 6w | 8w | 2.695* | 0.000 | 1.880 | 3.510 |
|  |  | 12w | 2.340* | 0.000 | 1.525 | 3.155 |
|  | 8w | 12w | -0.355 | 0.643 | -1.170 | 0.460 |
| Medium | 6w | 8w | 4.980* | 0.000 | 4.156 | 5.795 |
|  |  | 12w | 3.835* | 0.000 | 3.020 | 4.650 |
|  | 8w | 12w | -1.145* | 0.003 | -1.960 | -0.330 |
| High | 6w | 8w | 1.360* | 0.000 | 0.545 | 2.175 |
|  |  | 12w | -0.120 | 0.978 | -0.935 | 0.695 |
|  | 8w | 12w | -1.480* | 0.000 | -2.295 | -0.665 |
| ***Time*** | ***Group (I)*** | ***Group (J)*** |  |  |  |  |
| 6w | Control | Low | -1.910* | 0.000 | -2.812 | -1.008 |
|  |  | Medium | -4.173* | 0.000 | -5.076 | -3.271 |
|  |  | High | -0.522 | 0.542 | -1.424 | 0.381 |
|  | Low | Medium | -2.263* | 0.000 | -3.166 | -1.361 |
|  |  | High | 1.388* | 0.001 | 0.486 | 2.291 |
|  | Medium | High | 3.652* | 0.000 | 2.749 | 4.554 |
| 8w | Control | Low | 0.775 | 0.130 | -0.127 | 1.677 |
|  |  | Medium | 0.797 | 0.112 | -0.106 | 1.699 |
|  |  | High | 0.828 | 0.089 | -0.074 | 1.731 |
|  | Low | Medium | 0.022 | 1.000 | -0.881 | 0.924 |
|  |  | High | 0.053 | 1.000 | -0.849 | 0.956 |
|  | Medium | High | 0.032 | 1.000 | -0.871 | 0.934 |
| 12w | Control | Low | 0.388 | 0.819 | -0.514 | 1.291 |
|  |  | Medium | -0.380 | 0.833 | -1.282 | 0.522 |
|  |  | High | -0.683 | 0.237 | -1.586 | 0.219 |
|  | Low | Medium | -0.768 | 0.136 | -1.671 | 0.134 |
|  |  | High | -1.072* | 0.012 | -1.974 | -0.169 |
|  | Medium | High | -0.303 | 0.935 | -1.206 | 0.599 |
| 18w | Control | Long-High | -10.603* | 0.000 | -11.268 | -9.939 |
| #CVF%=Collagen area/Total muscle area×100%, collagen volume fraction (%) in Masson's trichrome staining. | | | | | | |
| *The mean difference is significant at the 0.05 level. | | | | | | |

| **Table S4. Simple effect analysis of pairwise comparisons between group and time in PAS** | | | | | | |
| --- | --- | --- | --- | --- | --- | --- |
| **AOD**# | | | **Mean Difference** | ***P*** | **95% Confidence Interval for Difference** | |
| ***Group*** | ***Time (I)*** | ***Time (J)*** | **(I－J)** |  | **Lower Bound** | **Upper Bound** |
| Control | 6w | 8w | 0.004 | 0.944 | -0.008 | 0.015 |
|  |  | 12w | 0.003 | 0.972 | -0.008 | 0.014 |
|  |  | 18w | 0.002 | 1.000 | -0.010 | 0.013 |
|  | 8w | 12w | -0.001 | 1.000 | -0.012 | 0.011 |
|  |  | 18w | -0.002 | 0.996 | -0.013 | 0.009 |
|  | 12w | 18w | -0.002 | 0.999 | -0.013 | 0.010 |
| Low | 6w | 8w | -0.040* | 0.000 | -0.050 | -0.029 |
|  |  | 12w | -0.055* | 0.000 | -0.065 | -0.045 |
|  | 8w | 12w | -0.015* | 0.002 | -0.025 | -0.005 |
| Medium | 6w | 8w | -0.004 | 0.658 | -0.015 | 0.006 |
|  |  | 12w | -0.056* | 0.000 | -0.066 | -0.046 |
|  | 8w | 12w | -0.052* | 0.000 | -0.062 | -0.042 |
| High | 6w | 8w | 0.016* | 0.001 | 0.006 | 0.026 |
|  |  | 12w | -0.020* | 0.000 | -0.031 | -0.010 |
|  | 8w | 12w | -0.037* | 0.000 | -0.047 | -0.026 |
| ***Time*** | ***Group (I)*** | ***Group (J)*** |  |  |  |  |
| 6w | Control | Low | 0.043* | 0.000 | 0.031 | 0.054 |
|  |  | Medium | 0.051* | 0.000 | 0.040 | 0.062 |
|  |  | High | 0.032* | 0.000 | 0.021 | 0.044 |
|  | Low | Medium | 0.008 | 0.281 | -0.003 | 0.019 |
|  |  | High | -0.010 | 0.089 | -0.022 | 0.001 |
|  | Medium | High | -0.019* | 0.000 | -0.030 | -0.007 |
| 8w | Control | Low | -0.001 | 1.000 | -0.012 | 0.011 |
|  |  | Medium | 0.043* | 0.000 | 0.032 | 0.054 |
|  |  | High | 0.045* | 0.000 | 0.034 | 0.056 |
|  | Low | Medium | 0.044* | 0.000 | 0.032 | 0.055 |
|  |  | High | 0.045* | 0.000 | 0.034 | 0.057 |
|  | Medium | High | 0.002 | 0.998 | -0.009 | 0.013 |
| 12w | Control | Low | -0.015* | 0.003 | -0.027 | -0.004 |
|  |  | Medium | -0.009 | 0.240 | -0.020 | 0.003 |
|  |  | High | 0.009 | 0.221 | -0.003 | 0.020 |
|  | Low | Medium | 0.007 | 0.485 | -0.004 | 0.018 |
|  |  | High | 0.024* | 0.000 | 0.013 | 0.035 |
|  | Medium | High | 0.017* | 0.001 | 0.006 | 0.028 |
| 18w | Control | Long-High | -0.028* | 0.000 | -0.037 | -0.020 |
| #AOD: Average optical density; PAS: Periodic Acid-Schiff stain. | | | | | | |
| *The mean difference is significant at the 0.05 level. | | | | | | |

| **Table S5. Simple effect analysis of pairwise comparisons between group and time in Sirius-red staining** | | | | | | |
| --- | --- | --- | --- | --- | --- | --- |
| **Col-I/Col-III^#^** | | | **Mean Difference** | ***P*** | **95% Confidence Interval for Difference** | |
| ***Group*** | ***Time (I)*** | ***Time (J)*** | **(I－J)** |  | **Lower Bound** | **Upper Bound** |
| Control | 6w | 8w | 0.007 | 1.000 | -0.706 | 0.720 |
|  |  | 12w | 0.004 | 1.000 | -0.709 | 0.717 |
|  |  | 18w | 0.006 | 1.000 | -0.707 | 0.719 |
|  | 8w | 12w | -0.003 | 1.000 | -0.716 | 0.710 |
|  |  | 18w | -0.001 | 1.000 | -0.714 | 0.712 |
|  | 12w | 18w | 0.002 | 1.000 | -0.711 | 0.715 |
| Low | 6w | 8w | -2.433* | 0.000 | -3.077 | -1.789 |
|  |  | 12w | -3.777* | 0.000 | -4.421 | -3.133 |
|  | 8w | 12w | -1.344* | 0.000 | -1.988 | -0.700 |
| Medium | 6w | 8w | 1.819* | 0.000 | 1.175 | 2.463 |
|  |  | 12w | 2.343* | 0.000 | 1.699 | 2.987 |
|  | 8w | 12w | 0.524 | 0.144 | -0.120 | 1.168 |
| High | 6w | 8w | 0.163 | 0.902 | -0.481 | 0.807 |
|  |  | 12w | -0.273 | 0.663 | -0.917 | 0.371 |
|  | 8w | 12w | -0.435 | 0.278 | -1.079 | 0.209 |
| ***Time*** | ***Group (I)*** | ***Group (J)*** |  |  |  |  |
| 6w | Control | Low | -0.670 | 0.076 | -1.383 | 0.043 |
|  |  | Medium | -2.426* | 0.000 | -3.139 | -1.713 |
|  |  | High | -0.168 | 0.989 | -0.881 | 0.545 |
|  | Low | Medium | -1.756* | 0.000 | -2.469 | -1.043 |
|  |  | High | 0.502 | 0.314 | -0.211 | 1.215 |
|  | Medium | High | 2.258* | 0.000 | 1.545 | 2.971 |
| 8w | Control | Low | -3.110* | 0.000 | -3.823 | -2.397 |
|  |  | Medium | -0.614 | 0.128 | -1.327 | 0.099 |
|  |  | High | -0.012 | 1.000 | -0.725 | 0.701 |
|  | Low | Medium | 2.496* | 0.000 | 1.783 | 3.208 |
|  |  | High | 3.097* | 0.000 | 2.384 | 3.810 |
|  | Medium | High | 0.602 | 0.143 | -0.111 | 1.315 |
| 12w | Control | Low | -4.450* | 0.000 | -5.163 | -3.738 |
|  |  | Medium | -0.087 | 1.000 | -0.800 | 0.626 |
|  |  | High | -0.444 | 0.454 | -1.157 | 0.268 |
|  | Low | Medium | 4.364* | 0.000 | 3.651 | 5.077 |
|  |  | High | 4.006* | 0.000 | 3.293 | 4.719 |
|  | Medium | High | -0.358 | 0.693 | -1.071 | 0.355 |
| 18w | Control | Long-High | -5.002* | 0.000 | -5.528 | -4.477 |
| #Ratio of Collagen-I area to Collagen-III area in Sirius-red staining. | | | | | | |
| *The mean difference is significant at the 0.05 level. | | | | | | |

| **Table S6. Simple effect analysis of pairwise comparisons between group and time in fast-twitch muscle fibers** | | | | | | |
| --- | --- | --- | --- | --- | --- | --- |
| **MYH1%^#^** | | | **Mean Difference** | ***P*** | **95% Confidence Interval for Difference** | |
| ***Group*** | ***Time (I)*** | ***Time (J)*** | **(I**－**J)** |  | **Lower Bound** | **Upper Bound** |
| Control | 6w | 8w | 0.455 | 1.000 | -3.175 | 4.085 |
|  |  | 12w | 0.126 | 1.000 | -3.504 | 3.756 |
|  |  | 18w | -0.989 | 0.976 | -4.619 | 2.641 |
|  | 8w | 12w | -0.329 | 1.000 | -3.959 | 3.301 |
|  |  | 18w | -1.443 | 0.867 | -5.074 | 2.187 |
|  | 12w | 18w | -1.114 | 0.957 | -4.745 | 2.516 |
| Low | 6w | 8w | -9.429* | 0.000 | -12.709 | -6.149 |
|  |  | 12w | -0.822 | 0.904 | -4.102 | 2.458 |
|  | 8w | 12w | 8.607* | 0.000 | 5.327 | 11.887 |
| Medium | 6w | 8w | -10.212* | 0.000 | -13.492 | -6.932 |
|  |  | 12w | -0.951 | 0.860 | -4.230 | 2.329 |
|  | 8w | 12w | 9.261* | 0.000 | 5.981 | 12.541 |
| High | 6w | 8w | -16.445* | 0.000 | -19.725 | -13.165 |
|  |  | 12w | -2.238 | 0.270 | -5.518 | 1.042 |
|  | 8w | 12w | 14.207* | 0.000 | 10.927 | 17.487 |
| ***Time*** | ***Group (I)*** | ***Group (J)*** |  |  |  |  |
| 6w | Control | Low | 18.618* | 0.000 | 14.988 | 22.248 |
|  |  | Medium | 17.991* | 0.000 | 14.361 | 21.621 |
|  |  | High | 21.849* | 0.000 | 18.219 | 25.479 |
|  | Low | Medium | -0.626 | 0.998 | -4.256 | 3.004 |
|  |  | High | 3.231 | 0.106 | -0.399 | 6.861 |
|  | Medium | High | 3.858* | 0.031 | 0.228 | 7.488 |
| 8w | Control | Low | 8.734* | 0.000 | 5.104 | 12.364 |
|  |  | Medium | 7.325* | 0.000 | 3.695 | 10.955 |
|  |  | High | 4.949* | 0.003 | 1.319 | 8.579 |
|  | Low | Medium | -1.409 | 0.879 | -5.039 | 2.221 |
|  |  | High | -3.785* | 0.037 | -7.415 | -0.155 |
|  | Medium | High | -2.376 | 0.397 | -6.006 | 1.254 |
| 12w | Control | Low | 17.670* | 0.000 | 14.040 | 21.300 |
|  |  | Medium | 16.915* | 0.000 | 13.285 | 20.545 |
|  |  | High | 19.485* | 0.000 | 15.855 | 23.115 |
|  | Low | Medium | -0.755 | 0.994 | -4.385 | 2.875 |
|  |  | High | 1.815 | 0.696 | -1.815 | 5.445 |
|  | Medium | High | 2.570 | 0.307 | -1.060 | 6.200 |
| 18w | Control | Long-High | -14.069* | 0.000 | -16.743 | -11.395 |
| #Percentage of MYH1-positive area in immunofluorescence staining of fast-twitch muscle fibers. | | | | | | |
| *The mean difference is significant at the 0.05 level. | | | | | | |

| **Table S7. Simple effect analysis of pairwise comparisons between group and time in slow-twitch muscle fibers** | | | | | | |
| --- | --- | --- | --- | --- | --- | --- |
| **MYH7%^#^** | | | **Mean Difference** | ***P*** | **95% Confidence Interval for Difference** | |
| ***Group*** | ***Time (I)*** | ***Time (J)*** | **(I－J)** |  | **Lower Bound** | **Upper Bound** |
| Control | 6w | 8w | 0.796 | 0.778 | -0.952 | 2.545 |
|  |  | 12w | 0.265 | 0.999 | -1.484 | 2.013 |
|  |  | 18w | 0.575 | 0.941 | -1.173 | 2.323 |
|  | 8w | 12w | -0.532 | 0.959 | -2.280 | 1.217 |
|  |  | 18w | -0.221 | 1.000 | -1.970 | 1.527 |
|  | 12w | 18w | 0.310 | 0.998 | -1.438 | 2.059 |
| Low | 6w | 8w | -9.269* | 0.000 | -10.849 | -7.689 |
|  |  | 12w | -14.787* | 0.000 | -16.367 | -13.208 |
|  | 8w | 12w | -5.518* | 0.000 | -7.098 | -3.939 |
| Medium | 6w | 8w | -3.735* | 0.000 | -5.314 | -2.155 |
|  |  | 12w | -12.840* | 0.000 | -14.420 | -11.260 |
|  | 8w | 12w | -9.105* | 0.000 | -10.685 | -7.526 |
| High | 6w | 8w | -0.435 | 0.877 | -2.015 | 1.144 |
|  |  | 12w | -35.192* | 0.000 | -36.772 | -33.613 |
|  | 8w | 12w | -34.757* | 0.000 | -36.336 | -33.177 |
| ***Time*** | ***Group (I)*** | ***Group (J)*** |  |  |  |  |
| 6w | Control | Low | 3.313* | 0.000 | 1.565 | 5.062 |
|  |  | Medium | 3.113* | 0.000 | 1.365 | 4.861 |
|  |  | High | 2.499* | 0.001 | 0.750 | 4.247 |
|  | Low | Medium | -0.200 | 1.000 | -1.949 | 1.548 |
|  |  | High | -0.815 | 0.759 | -2.563 | 0.934 |
|  | Medium | High | -0.614 | 0.921 | -2.362 | 1.134 |
| 8w | Control | Low | -6.752* | 0.000 | -8.500 | -5.004 |
|  |  | Medium | -1.418 | 0.174 | -3.166 | 0.330 |
|  |  | High | 1.267 | 0.282 | -0.481 | 3.015 |
|  | Low | Medium | 5.334* | 0.000 | 3.586 | 7.082 |
|  |  | High | 8.019* | 0.000 | 6.271 | 9.767 |
|  | Medium | High | 2.685* | 0.001 | 0.937 | 4.433 |
| 12w | Control | Low | -11.739* | 0.000 | -13.487 | -9.990 |
|  |  | Medium | -9.992* | 0.000 | -11.740 | -8.244 |
|  |  | High | -32.958* | 0.000 | -34.706 | -31.210 |
|  | Low | Medium | 1.747 | 0.050 | -0.001 | 3.495 |
|  |  | High | -21.220* | 0.000 | -22.968 | -19.471 |
|  | Medium | High | -22.966* | 0.000 | -24.715 | -21.218 |
| 18w | Control | Long-High | -12.558* | 0.000 | -13.846 | -11.270 |
| #Percentage of MYH7-positive area in immunofluorescence staining of slow-twitch muscle fibers. | | | | | | |
| *The mean difference is significant at the 0.05 level. | | | | | | |

| **Table S8. Simple effect analysis of pairwise comparisons between group and time in Ki-67 staining** | | | | | | |
| --- | --- | --- | --- | --- | --- | --- |
| **Ki-67^+^ cells%^#^** | | | **Mean Difference** | ***P*** | **95% Confidence Interval for Difference** | |
| ***Group*** | ***Time (I)*** | ***Time (J)*** | **(I－J)** |  | **Lower Bound** | **Upper Bound** |
| Control | 6w | 8w | -0.732 | 0.945 | -2.987 | 1.523 |
|  |  | 12w | 0.220 | 1.000 | -2.036 | 2.475 |
|  |  | 18w | 0.171 | 1.000 | -2.085 | 2.426 |
|  | 8w | 12w | 0.952 | 0.832 | -1.304 | 3.207 |
|  |  | 18w | 0.903 | 0.863 | -1.353 | 3.158 |
|  | 12w | 18w | -0.049 | 1.000 | -2.304 | 2.206 |
| Low | 6w | 8w | -9.486* | 0.000 | -11.524 | -7.448 |
|  |  | 12w | 4.998* | 0.000 | 2.961 | 7.036 |
|  | 8w | 12w | 14.484* | 0.000 | 12.447 | 16.522 |
| Medium | 6w | 8w | -6.715* | 0.000 | -8.753 | -4.677 |
|  |  | 12w | 19.023* | 0.000 | 16.986 | 21.061 |
|  | 8w | 12w | 25.738* | 0.000 | 23.701 | 27.776 |
| High | 6w | 8w | 10.617* | 0.000 | 8.579 | 12.654 |
|  |  | 12w | 12.567* | 0.000 | 10.529 | 14.605 |
|  | 8w | 12w | 1.950 | 0.065 | -0.087 | 3.988 |
| ***Time*** | ***Group (I)*** | ***Group (J)*** |  |  |  |  |
| 6w | Control | Low | -11.455* | 0.000 | -13.710 | -9.199 |
|  |  | Medium | -20.193* | 0.000 | -22.448 | -17.937 |
|  |  | High | -15.720* | 0.000 | -17.975 | -13.464 |
|  | Low | Medium | -8.738* | 0.000 | -10.993 | -6.482 |
|  |  | High | -4.265* | 0.000 | -6.520 | -2.010 |
|  | Medium | High | 4.473* | 0.000 | 2.217 | 6.728 |
| 8w | Control | Low | -20.209* | 0.000 | -22.464 | -17.953 |
|  |  | Medium | -26.176* | 0.000 | -28.431 | -23.920 |
|  |  | High | -4.371* | 0.000 | -6.627 | -2.116 |
|  | Low | Medium | -5.967* | 0.000 | -8.222 | -3.712 |
|  |  | High | 15.838* | 0.000 | 13.582 | 18.093 |
|  | Medium | High | 21.805* | 0.000 | 19.549 | 24.060 |
| 12w | Control | Low | -6.676* | 0.000 | -8.931 | -4.421 |
|  |  | Medium | -1.389 | 0.468 | -3.644 | 0.866 |
|  |  | High | -3.372* | 0.001 | -5.628 | -1.117 |
|  | Low | Medium | 5.287* | 0.000 | 3.032 | 7.542 |
|  |  | High | 3.304* | 0.001 | 1.048 | 5.559 |
|  | Medium | High | -1.984 | 0.114 | -4.239 | 0.272 |
| 18w | Control | Long-High | 3.374* | 0.000 | 1.712 | 5.035 |
| #Percentage of MKI67-positive cells in Ki-67 immunofluorescence staining. | | | | | | |
| *The mean difference is significant at the 0.05 level. | | | | | | |

| **Table S9. Simple effect analysis of pairwise comparisons between group and time in TUNEL assay** | | | | | | |
| --- | --- | --- | --- | --- | --- | --- |
| **Apoptotic cells%^#^** | | | **Mean Difference** | ***P*** | **95% Confidence Interval for Difference** | |
| ***Group*** | ***Time (I)*** | ***Time (J)*** | **(I－J)** |  | **Lower Bound** | **Upper Bound** |
| Control | 6w | 8w | 0.122 | 1.000 | -6.040 | 6.285 |
|  |  | 12w | 0.404 | 1.000 | -5.758 | 6.567 |
|  |  | 18w | 0.492 | 1.000 | -5.670 | 6.655 |
|  | 8w | 12w | 0.282 | 1.000 | -5.881 | 6.444 |
|  |  | 18w | 0.370 | 1.000 | -5.793 | 6.532 |
|  | 12w | 18w | 0.088 | 1.000 | -6.074 | 6.251 |
| Low | 6w | 8w | -8.364* | 0.001 | -13.932 | -2.796 |
|  |  | 12w | -23.725* | 0.000 | -29.293 | -18.157 |
|  | 8w | 12w | -15.361* | 0.000 | -20.929 | -9.793 |
| Medium | 6w | 8w | -4.095 | 0.212 | -9.663 | 1.473 |
|  |  | 12w | -14.623* | 0.000 | -20.191 | -9.055 |
|  | 8w | 12w | -10.528* | 0.000 | -16.096 | -4.960 |
| High | 6w | 8w | -7.731* | 0.003 | -13.298 | -2.163 |
|  |  | 12w | -11.939* | 0.000 | -17.507 | -6.371 |
|  | 8w | 12w | -4.209 | 0.192 | -9.777 | 1.359 |
| ***Time*** | ***Group (I)*** | ***Group (J)*** |  |  |  |  |
| 6w | Control | Low | -14.359* | 0.000 | -20.522 | -8.197 |
|  |  | Medium | -28.993* | 0.000 | -35.155 | -22.830 |
|  |  | High | -38.695* | 0.000 | -44.857 | -32.532 |
|  | Low | Medium | -14.634* | 0.000 | -20.796 | -8.471 |
|  |  | High | -24.335* | 0.000 | -30.498 | -18.173 |
|  | Medium | High | -9.702* | 0.000 | -15.864 | -3.539 |
| 8w | Control | Low | -22.846* | 0.000 | -29.008 | -16.683 |
|  |  | Medium | -33.211* | 0.000 | -39.373 | -27.048 |
|  |  | High | -46.548* | 0.000 | -52.710 | -40.385 |
|  | Low | Medium | -10.365* | 0.000 | -16.527 | -4.202 |
|  |  | High | -23.702* | 0.000 | -29.864 | -17.539 |
|  | Medium | High | -13.337* | 0.000 | -19.500 | -7.174 |
| 12w | Control | Low | -38.489* | 0.000 | -44.651 | -32.326 |
|  |  | Medium | -44.020* | 0.000 | -50.183 | -37.857 |
|  |  | High | -51.038* | 0.000 | -57.201 | -44.876 |
|  | Low | Medium | -5.531 | 0.101 | -11.694 | 0.631 |
|  |  | High | -12.550* | 0.000 | -18.712 | -6.387 |
|  | Medium | High | -7.018* | 0.017 | -13.181 | -0.856 |
| 18w | Control | Long-High | -23.148* | 0.000 | -27.688 | -18.609 |
| #Percentage of apoptotic cells in TdT-mediated dUTP nick-end labelling (TUNEL) assay. | | | | | | |
| *The mean difference is significant at the 0.05 level. | | | | | | |

| **Table S10. Simple effect analysis of pairwise comparisons between group and time in myoblasts** | | | | | | |
| --- | --- | --- | --- | --- | --- | --- |
| **MyoD^+^Desmin^+^ cells%^#^** | | | **Mean Difference** | ***P*** | **95% Confidence Interval for Difference** | |
| ***Group*** | ***Time (I)*** | ***Time (J)*** | **(I－J)** |  | **Lower Bound** | **Upper Bound** |
| Control | 6w | 8w | 1.142 | 0.958 | -2.590 | 4.874 |
|  |  | 12w | 0.296 | 1.000 | -3.436 | 4.028 |
|  |  | 18w | 0.962 | 0.982 | -2.770 | 4.693 |
|  | 8w | 12w | -0.846 | 0.991 | -4.578 | 2.886 |
|  |  | 18w | -0.180 | 1.000 | -3.912 | 3.551 |
|  | 12w | 18w | 0.666 | 0.997 | -3.066 | 4.397 |
| Low | 6w | 8w | -12.056* | 0.000 | -15.428 | -8.685 |
|  |  | 12w | -5.351* | 0.001 | -8.722 | -1.979 |
|  | 8w | 12w | 6.706* | 0.000 | 3.334 | 10.077 |
| Medium | 6w | 8w | -11.993* | 0.000 | -15.364 | -8.621 |
|  |  | 12w | -5.201* | 0.001 | -8.572 | -1.830 |
|  | 8w | 12w | 6.792* | 0.000 | 3.420 | 10.163 |
| High | 6w | 8w | -12.125* | 0.000 | -15.496 | -8.754 |
|  |  | 12w | -4.328* | 0.007 | -7.699 | -0.956 |
|  | 8w | 12w | 7.797* | 0.000 | 4.426 | 11.169 |
| ***Time*** | ***Group (I)*** | ***Group (J)*** |  |  |  |  |
| 6w | Control | Low | -2.405 | 0.415 | -6.137 | 1.327 |
|  |  | Medium | -4.659* | 0.007 | -8.390 | -0.927 |
|  |  | High | -7.295* | 0.000 | -11.027 | -3.564 |
|  | Low | Medium | -2.254 | 0.491 | -5.985 | 1.478 |
|  |  | High | -4.890* | 0.004 | -8.622 | -1.159 |
|  | Medium | High | -2.636 | 0.309 | -6.368 | 1.095 |
| 8w | Control | Low | -15.603* | 0.000 | -19.335 | -11.872 |
|  |  | Medium | -17.793* | 0.000 | -21.525 | -14.062 |
|  |  | High | -20.562* | 0.000 | -24.294 | -16.831 |
|  | Low | Medium | -2.190 | 0.525 | -5.922 | 1.542 |
|  |  | High | -4.959* | 0.004 | -8.691 | -1.227 |
|  | Medium | High | -2.769 | 0.257 | -6.501 | 0.963 |
| 12w | Control | Low | -8.052* | 0.000 | -11.783 | -4.320 |
|  |  | Medium | -10.156* | 0.000 | -13.887 | -6.424 |
|  |  | High | -11.919* | 0.000 | -15.650 | -8.187 |
|  | Low | Medium | -2.104 | 0.570 | -5.836 | 1.627 |
|  |  | High | -3.867* | 0.038 | -7.599 | -0.136 |
|  | Medium | High | -1.763 | 0.748 | -5.495 | 1.969 |
| 18w | Control | Long-High | -34.237* | 0.000 | -36.986 | -31.488 |
| #Percentage of MyoD^+^Desmin^+^ cells in immunofluorescence staining of myoblasts. | | | | | | |
| *The mean difference is significant at the 0.05 level. | | | | | | |

| **Table S11. Simple effect analysis of pairwise comparisons between group and time in IL-1β immunohistochemistry** | | | | | | |
| --- | --- | --- | --- | --- | --- | --- |
| **AOD^#^** | | | **Mean Difference** | ***P*** | **95% Confidence Interval for Difference** | |
| ***Group*** | ***Time (I)*** | ***Time (J)*** | **(I－J)** |  | **Lower Bound** | **Upper Bound** |
| Control | 6w | 8w | 0.004 | 0.999 | -0.028 | 0.036 |
|  |  | 12w | 0.003 | 1.000 | -0.029 | 0.035 |
|  |  | 18w | 0.001 | 1.000 | -0.030 | 0.033 |
|  | 8w | 12w | -0.001 | 1.000 | -0.033 | 0.031 |
|  |  | 18w | -0.003 | 1.000 | -0.035 | 0.029 |
|  | 12w | 18w | -0.001 | 1.000 | -0.033 | 0.030 |
| Low | 6w | 8w | 0.066* | 0.000 | 0.037 | 0.095 |
|  |  | 12w | -0.026 | 0.090 | -0.055 | 0.003 |
|  | 8w | 12w | -0.092* | 0.000 | -0.121 | -0.063 |
| Medium | 6w | 8w | -0.036* | 0.010 | -0.065 | -0.007 |
|  |  | 12w | -0.061* | 0.000 | -0.089 | -0.032 |
|  | 8w | 12w | -0.024 | 0.120 | -0.053 | 0.004 |
| High | 6w | 8w | 0.003 | 0.993 | -0.026 | 0.032 |
|  |  | 12w | -0.037* | 0.007 | -0.066 | -0.009 |
|  | 8w | 12w | -0.040* | 0.003 | -0.069 | -0.011 |
| ***Time*** | ***Group (I)*** | ***Group (J)*** |  |  |  |  |
| 6w | Control | Low | -0.050* | 0.000 | -0.082 | -0.018 |
|  |  | Medium | -0.034* | 0.032 | -0.066 | -0.002 |
|  |  | High | -0.058* | 0.000 | -0.090 | -0.026 |
|  | Low | Medium | 0.016 | 0.695 | -0.016 | 0.048 |
|  |  | High | -0.008 | 0.981 | -0.040 | 0.024 |
|  | Medium | High | -0.024 | 0.232 | -0.056 | 0.008 |
| 8w | Control | Low | 0.012 | 0.901 | -0.020 | 0.044 |
|  |  | Medium | -0.074* | 0.000 | -0.106 | -0.042 |
|  |  | High | -0.060* | 0.000 | -0.092 | -0.028 |
|  | Low | Medium | -0.086* | 0.000 | -0.118 | -0.054 |
|  |  | High | -0.072* | 0.000 | -0.103 | -0.040 |
|  | Medium | High | 0.014 | 0.781 | -0.017 | 0.046 |
| 12w | Control | Low | -0.079* | 0.000 | -0.111 | -0.047 |
|  |  | Medium | -0.097* | 0.000 | -0.129 | -0.065 |
|  |  | High | -0.099* | 0.000 | -0.131 | -0.067 |
|  | Low | Medium | -0.018 | 0.541 | -0.050 | 0.013 |
|  |  | High | -0.020 | 0.459 | -0.052 | 0.012 |
|  | Medium | High | -0.001 | 1.000 | -0.033 | 0.031 |
| 18w | Control | Long-High | 0.033* | 0.007 | 0.009 | 0.056 |
| #AOD: Average optical density; IL-1β: Interleukin-1β. | | | | | | |
| *The mean difference is significant at the 0.05 level. | | | | | | |

| **Table S12. Simple effect analysis of pairwise comparisons between group and time in IL-6 immunohistochemistry** | | | | | | |
| --- | --- | --- | --- | --- | --- | --- |
| **AOD^#^** | | | **Mean Difference** | ***P*** | **95% Confidence Interval for Difference** | |
| ***Group*** | ***Time (I)*** | ***Time (J)*** | **(I－J)** |  | **Lower Bound** | **Upper Bound** |
| Control | 6w | 8w | 5.000×10^-6^ | 1.000 | -0.002 | 0.002 |
|  |  | 12w | -1.333×10^-5^ | 1.000 | -0.002 | 0.002 |
|  |  | 18w | 1.167×10^-5^ | 1.000 | -0.002 | 0.002 |
|  | 8w | 12w | -1.833×10^-5^ | 1.000 | -0.002 | 0.002 |
|  |  | 18w | 6.667×10^-6^ | 1.000 | -0.002 | 0.002 |
|  | 12w | 18w | 2.500×10^-5^ | 1.000 | -0.002 | 0.002 |
| Low | 6w | 8w | 0.004* | 0.000 | 0.002 | 0.006 |
|  |  | 12w | 0.008* | 0.000 | 0.007 | 0.010 |
|  | 8w | 12w | 0.004* | 0.000 | 0.003 | 0.006 |
| Medium | 6w | 8w | 0.020* | 0.000 | -0.022 | -0.018 |
|  |  | 12w | 0.006* | 0.000 | 0.004 | 0.008 |
|  | 8w | 12w | 0.026* | 0.000 | 0.024 | 0.028 |
| High | 6w | 8w | -0.005* | 0.000 | -0.006 | -0.003 |
|  |  | 12w | -0.002 | 0.098 | 0.003 | 0.000 |
|  | 8w | 12w | 0.003* | 0.000 | 0.001 | 0.005 |
| ***Time*** | ***Group (I)*** | ***Group (J)*** |  |  |  |  |
| 6w | Control | Low | -0.008* | 0.000 | -0.010 | -0.006 |
|  |  | Medium | -0.007* | 0.000 | -0.009 | -0.005 |
|  |  | High | -0.007* | 0.000 | -0.009 | -0.005 |
|  | Low | Medium | 0.001 | 0.574 | -0.001 | 0.003 |
|  |  | High | 0.001 | 0.559 | -0.001 | 0.003 |
|  | Medium | High | 1.500×10^-5^ | 1.000 | -0.002 | 0.002 |
| 8w | Control | Low | -0.004* | 0.000 | -0.006 | -0.002 |
|  |  | Medium | -0.027* | 0.000 | -0.029 | -0.025 |
|  |  | High | -0.012* | 0.000 | -0.014 | -0.010 |
|  | Low | Medium | -0.023* | 0.000 | -0.025 | -0.021 |
|  |  | High | -0.007* | 0.000 | -0.009 | -0.006 |
|  | Medium | High | 0.015* | 0.000 | 0.013 | 0.017 |
| 12w | Control | Low | -8.167×10^-5^ | 1.000 | -0.002 | 0.002 |
|  |  | Medium | -0.001 | 0.611 | -0.003 | -0.001 |
|  |  | High | -0.009* | 0.000 | -0.011 | -0.007 |
|  | Low | Medium | -0.001 | 0.692 | -0.003 | 0.001 |
|  |  | High | -0.009* | 0.000 | -0.011 | -0.007 |
|  | Medium | High | -0.008* | 0.000 | -0.010 | -0.006 |
| 18w | Control | Long-High | 0.000 | 0.871 | -0.002 | 0.001 |
| #AOD: Average optical density; IL-6: Interleukin-6. | | | | | | |
| *The mean difference is significant at the 0.05 level. | | | | | | |

| **Table S13. Simple effect analysis of pairwise comparisons between group and time in TNF-α immunohistochemistry** | | | | | | |
| --- | --- | --- | --- | --- | --- | --- |
| **AOD^#^** | | | **Mean Difference** | ***P*** | **95% Confidence Interval for Difference** | |
| ***Group*** | ***Time (I)*** | ***Time (J)*** | **(I－J)** |  | **Lower Bound** | **Upper Bound** |
| Control | 6w | 8w | 0.001 | 1.000 | -0.007 | 0.009 |
|  |  | 12w | 0.003 | 0.858 | -0.005 | 0.012 |
|  |  | 18w | 0.002 | 0.960 | -0.006 | 0.011 |
|  | 8w | 12w | 0.002 | 0.971 | -0.006 | 0.011 |
|  |  | 18w | 0.001 | 0.997 | -0.007 | 0.010 |
|  | 12w | 18w | -0.001 | 1.000 | -0.009 | 0.007 |
| Low | 6w | 8w | -0.003 | 0.623 | -0.011 | 0.004 |
|  |  | 12w | 0.020* | 0.000 | 0.013 | 0.027 |
|  | 8w | 12w | 0.023* | 0.000 | 0.016 | 0.031 |
| Medium | 6w | 8w | 0.007 | 0.072 | 0.000 | 0.014 |
|  |  | 12w | 0.023* | 0.000 | 0.016 | 0.030 |
|  | 8w | 12w | 0.016* | 0.000 | 0.009 | 0.023 |
| High | 6w | 8w | 0.004 | 0.406 | -0.003 | 0.012 |
|  |  | 12w | 0.029* | 0.000 | 0.021 | 0.036 |
|  | 8w | 12w | 0.024* | 0.000 | 0.017 | 0.032 |
| ***Time*** | ***Group (I)*** | ***Group (J)*** |  |  |  |  |
| 6w | Control | Low | -0.014* | 0.000 | -0.022 | -0.006 |
|  |  | Medium | -0.041* | 0.000 | -0.049 | -0.033 |
|  |  | High | -0.056* | 0.000 | -0.065 | -0.048 |
|  | Low | Medium | -0.027* | 0.000 | -0.035 | -0.019 |
|  |  | High | -0.042* | 0.000 | -0.051 | -0.034 |
|  | Medium | High | -0.015* | 0.000 | -0.024 | -0.007 |
| 8w | Control | Low | -0.018* | 0.000 | -0.027 | -0.010 |
|  |  | Medium | -0.035* | 0.000 | -0.043 | -0.027 |
|  |  | High | -0.053* | 0.000 | -0.061 | -0.045 |
|  | Low | Medium | -0.017* | 0.000 | -0.025 | -0.009 |
|  |  | High | -0.035* | 0.000 | -0.043 | -0.027 |
|  | Medium | High | -0.018* | 0.000 | -0.026 | -0.010 |
| 12w | Control | Low | 0.003 | 0.946 | -0.006 | 0.011 |
|  |  | Medium | -0.022* | 0.000 | -0.030 | -0.013 |
|  |  | High | -0.031* | 0.000 | -0.039 | -0.023 |
|  | Low | Medium | -0.024* | 0.000 | -0.032 | -0.016 |
|  |  | High | -0.034* | 0.000 | -0.042 | -0.026 |
|  | Medium | High | -0.010* | 0.013 | -0.018 | -0.001 |
| 18w | Control | Long-High | -0.024* | 0.000 | -0.030 | -0.018 |
| #AOD: Average optical density; TNF-α: Tumor necrosis factor-α. | | | | | | |
| *The mean difference is significant at the 0.05 level. | | | | | | |

| **Table S14. Simple effect analysis of pairwise comparisons between group and time in monocytes** | | | | | | |
| --- | --- | --- | --- | --- | --- | --- |
| **CD68^+^ cells%^#^** | | | **Mean Difference** | ***P*** | **95% Confidence Interval for Difference** | |
| ***Group*** | ***Time (I)*** | ***Time (J)*** | **(I－J)** |  | **Lower Bound** | **Upper Bound** |
| Control | 6w | 8w | -0.171 | 1.000 | -1.784 | 1.441 |
|  |  | 12w | -0.075 | 1.000 | -1.687 | 1.538 |
|  |  | 18w | -0.078 | 1.000 | -1.691 | 1.534 |
|  | 8w | 12w | 0.097 | 1.000 | -1.516 | 1.709 |
|  |  | 18w | 0.093 | 1.000 | -1.520 | 1.706 |
|  | 12w | 18w | -0.004 | 1.000 | -1.617 | 1.609 |
| Low | 6w | 8w | -4.058* | 0.000 | -5.515 | -2.601 |
|  |  | 12w | -12.132* | 0.000 | -13.589 | -10.675 |
|  | 8w | 12w | -8.074* | 0.000 | -9.531 | -6.617 |
| Medium | 6w | 8w | 16.768* | 0.000 | 15.311 | 18.225 |
|  |  | 12w | 8.946* | 0.000 | 7.488 | 10.403 |
|  | 8w | 12w | -7.822* | 0.000 | -9.279 | -6.365 |
| High | 6w | 8w | -1.907* | 0.006 | -3.364 | -0.450 |
|  |  | 12w | -7.532* | 0.000 | -8.990 | -6.075 |
|  | 8w | 12w | -5.626* | 0.000 | -7.083 | -4.169 |
| ***Time*** | ***Group (I)*** | ***Group (J)*** |  |  |  |  |
| 6w | Control | Low | -1.829* | 0.018 | -3.442 | -0.216 |
|  |  | Medium | -24.394* | 0.000 | -26.007 | -22.782 |
|  |  | High | -10.292* | 0.000 | -11.904 | -8.679 |
|  | Low | Medium | -22.565* | 0.000 | -24.178 | -20.952 |
|  |  | High | -8.463* | 0.000 | -10.075 | -6.850 |
|  | Medium | High | 14.103* | 0.000 | 12.490 | 15.715 |
| 8w | Control | Low | -5.716* | 0.000 | -7.329 | -4.103 |
|  |  | Medium | -7.455* | 0.000 | -9.068 | -5.843 |
|  |  | High | -12.027* | 0.000 | -13.640 | -10.414 |
|  | Low | Medium | -1.739* | 0.028 | -3.352 | -0.127 |
|  |  | High | -6.311* | 0.000 | -7.924 | -4.698 |
|  | Medium | High | -4.572* | 0.000 | -6.185 | -2.959 |
| 12w | Control | Low | -13.887* | 0.000 | -15.500 | -12.274 |
|  |  | Medium | -15.374* | 0.000 | -16.987 | -13.761 |
|  |  | High | -17.749* | 0.000 | -19.362 | -16.137 |
|  | Low | Medium | -1.487 | 0.086 | -3.100 | 0.125 |
|  |  | High | -3.863* | 0.000 | -5.475 | -2.250 |
|  | Medium | High | -2.375* | 0.001 | -3.988 | -0.763 |
| 18w | Control | Long-High | -20.079* | 0.000 | -21.267 | -18.890 |
| #Percentage of CD68^+^ cells in immunofluorescence staining of monocytes. | | | | | | |
| *The mean difference is significant at the 0.05 level. | | | | | | |

| **Table S15. Simple effect analysis of pairwise comparisons between group and time in M1 macrophages** | | | | | | |
| --- | --- | --- | --- | --- | --- | --- |
| **CD68^+^iNOS^+^ cells%^#^** | | | **Mean Difference** | ***P*** | **95% Confidence Interval for Difference** | |
| ***Group*** | ***Time (I)*** | ***Time (J)*** | **(I－J)** |  | **Lower Bound** | **Upper Bound** |
| Control | 6w | 8w | 0.043 | 0.998 | -0.204 | 0.289 |
|  |  | 12w | 0.030 | 1.000 | -0.217 | 0.276 |
|  |  | 18w | 0.023 | 1.000 | -0.224 | 0.269 |
|  | 8w | 12w | -0.013 | 1.000 | -0.259 | 0.233 |
|  |  | 18w | -0.020 | 1.000 | -0.267 | 0.226 |
|  | 12w | 18w | -0.007 | 1.000 | -0.254 | 0.239 |
| Low | 6w | 8w | 0.053 | 0.915 | -0.169 | 0.276 |
|  |  | 12w | -0.021 | 0.994 | -0.243 | 0.202 |
|  | 8w | 12w | -0.074 | 0.805 | -0.296 | 0.149 |
| Medium | 6w | 8w | 3.533* | 0.000 | 3.310 | 3.755 |
|  |  | 12w | 3.543* | 0.000 | 3.321 | 3.766 |
|  | 8w | 12w | 0.010 | 0.999 | -0.212 | 0.233 |
| High | 6w | 8w | 0.990* | 0.000 | 0.768 | 1.213 |
|  |  | 12w | 0.930* | 0.000 | 0.708 | 1.153 |
|  | 8w | 12w | -0.060 | 0.884 | -0.283 | 0.163 |
| ***Time*** | ***Group (I)*** | ***Group (J)*** |  |  |  |  |
| 6w | Control | Low | 0.010 | 1.000 | -0.236 | 0.256 |
|  |  | Medium | -3.514* | 0.000 | -3.761 | -3.268 |
|  |  | High | -0.955* | 0.000 | -1.201 | -0.708 |
|  | Low | Medium | -3.524* | 0.000 | -3.771 | -3.278 |
|  |  | High | -0.965* | 0.000 | -1.211 | -0.718 |
|  | Medium | High | 2.560* | 0.000 | 2.314 | 2.806 |
| 8w | Control | Low | 0.021 | 1.000 | -0.226 | 0.267 |
|  |  | Medium | -0.024 | 1.000 | -0.271 | 0.222 |
|  |  | High | -0.007 | 1.000 | -0.253 | 0.240 |
|  | Low | Medium | -0.045 | 0.997 | -0.291 | 0.202 |
|  |  | High | -0.027 | 1.000 | -0.274 | 0.219 |
|  | Medium | High | 0.018 | 1.000 | -0.229 | 0.264 |
| 12w | Control | Low | -0.040 | 0.998 | -0.287 | 0.206 |
|  |  | Medium | -0.001 | 1.000 | -0.247 | 0.246 |
|  |  | High | -0.054 | 0.992 | -0.300 | 0.193 |
|  | Low | Medium | 0.039 | 0.999 | -0.207 | 0.286 |
|  |  | High | -0.013 | 1.000 | -0.260 | 0.233 |
|  | Medium | High | -0.053 | 0.993 | -0.299 | 0.193 |
| 18w | Control | Long-High | -0.033 | 0.719 | -0.214 | 0.149 |
| #Percentage of CD68^+^iNOS^+^ cells in immunofluorescence staining of M1 macrophages. | | | | | | |
| *The mean difference is significant at the 0.05 level. | | | | | | |

| **Table S16. Simple effect analysis of pairwise comparisons between group and time in M2 macrophages** | | | | | | |
| --- | --- | --- | --- | --- | --- | --- |
| **CD163^+^CD206^+^ cells%^#^** | | | **Mean Difference** | ***P*** | **95% Confidence Interval for Difference** | |
| ***Group*** | ***Time (I)*** | ***Time (J)*** | **(I－J)** |  | **Lower Bound** | **Upper Bound** |
| Control | 6w | 8w | -0.047 | 1.000 | -1.505 | 1.412 |
|  |  | 12w | 0.043 | 1.000 | -1.416 | 1.501 |
|  |  | 18w | 0.141 | 1.000 | -1.317 | 1.600 |
|  | 8w | 12w | 0.089 | 1.000 | -1.369 | 1.548 |
|  |  | 18w | 0.188 | 1.000 | -1.270 | 1.647 |
|  | 12w | 18w | 0.099 | 1.000 | -1.360 | 1.558 |
| Low | 6w | 8w | -2.362* | 0.000 | -3.680 | -1.044 |
|  |  | 12w | -5.289* | 0.000 | -6.606 | -3.971 |
|  | 8w | 12w | -2.927* | 0.000 | -4.244 | -1.609 |
| Medium | 6w | 8w | 15.619* | 0.000 | 14.301 | 16.937 |
|  |  | 12w | 14.124* | 0.000 | 12.806 | 15.442 |
|  | 8w | 12w | -1.495* | 0.021 | -2.813 | -0.177 |
| High | 6w | 8w | 6.777* | 0.000 | 5.459 | 8.095 |
|  |  | 12w | 2.692* | 0.000 | 1.375 | 4.010 |
|  | 8w | 12w | -4.085* | 0.000 | -5.402 | -2.767 |
| ***Time*** | ***Group (I)*** | ***Group (J)*** |  |  |  |  |
| 6w | Control | Low | 1.217 | 0.152 | -0.242 | 2.675 |
|  |  | Medium | -13.303* | 0.000 | -14.762 | -11.845 |
|  |  | High | -0.280 | 0.996 | -1.738 | 1.179 |
|  | Low | Medium | -14.520* | 0.000 | -15.979 | -13.061 |
|  |  | High | -1.496* | 0.041 | -2.955 | -0.038 |
|  | Medium | High | 13.024* | 0.000 | 11.565 | 14.482 |
| 8w | Control | Low | -1.098 | 0.242 | -2.557 | 0.360 |
|  |  | Medium | 2.362* | 0.000 | 0.904 | 3.821 |
|  |  | High | 6.544* | 0.000 | 5.085 | 8.003 |
|  | Low | Medium | 3.461* | 0.000 | 2.002 | 4.919 |
|  |  | High | 7.643* | 0.000 | 6.184 | 9.101 |
|  | Medium | High | 4.182* | 0.000 | 2.723 | 5.640 |
| 12w | Control | Low | -4.114* | 0.000 | -5.573 | -2.656 |
|  |  | Medium | 0.778 | 0.631 | -0.680 | 2.237 |
|  |  | High | 2.370* | 0.000 | 0.912 | 3.829 |
|  | Low | Medium | 4.893* | 0.000 | 3.434 | 6.351 |
|  |  | High | 6.484* | 0.000 | 5.026 | 7.943 |
|  | Medium | High | 1.592* | 0.025 | 0.133 | 3.051 |
| 18w | Control | Long-High | -1.812* | 0.001 | -2.886 | -0.737 |
| #Percentage of CD163^+^CD206^+^ cells in immunofluorescence staining of M2 macrophages. | | | | | | |
| *The mean difference is significant at the 0.05 level. | | | | | | |

| **Table S17. Simple effect analysis of pairwise comparisons between group and time in intermuscular vessels** | | | | | | |
| --- | --- | --- | --- | --- | --- | --- |
| **Vascular density^#^** | | | **Mean Difference** | ***P*** | **95% Confidence Interval for Difference** | |
| ***Group*** | ***Time (I)*** | ***Time (J)*** | **(I－J)** |  | **Lower Bound** | **Upper Bound** |
| Control | 6w | 8w | -2.220×10^-16^ | 1.000 | -0.100 | 0.100 |
|  |  | 12w | 5.551×10^-16^ | 1.000 | -0.100 | 0.100 |
|  |  | 18w | 1.998×10^-15^ | 1.000 | -0.100 | 0.100 |
|  | 8w | 12w | 7.636×10^-16^ | 1.000 | -0.100 | 0.100 |
|  |  | 18w | 2.220×10^-15^ | 1.000 | -0.100 | 0.100 |
|  | 12w | 18w | 1.457×10^-15^ | 1.000 | -0.100 | 0.100 |
| Low | 6w | 8w | -1.407* | 0.000 | -1.497 | -1.316 |
|  |  | 12w | -1.593* | 0.000 | -1.684 | -1.503 |
|  | 8w | 12w | -0.187* | 0.000 | -0.277 | -0.096 |
| Medium | 6w | 8w | -0.857* | 0.000 | -0.947 | -0.766 |
|  |  | 12w | -0.448* | 0.000 | -0.539 | -0.358 |
|  | 8w | 12w | 0.408* | 0.000 | 0.318 | 0.499 |
| High | 6w | 8w | -0.955* | 0.000 | -1.045 | -0.865 |
|  |  | 12w | -0.158* | 0.000 | -0.249 | -0.068 |
|  | 8w | 12w | 0.797* | 0.000 | 0.706 | 0.887 |
| ***Time*** | ***Group (I)*** | ***Group (J)*** |  |  |  |  |
| 6w | Control | Low | -0.102* | 0.044 | -0.201 | -0.002 |
|  |  | Medium | -0.150* | 0.001 | -0.250 | -0.050 |
|  |  | High | -0.240* | 0.000 | -0.340 | -0.140 |
|  | Low | Medium | -0.048 | 0.726 | -0.148 | 0.051 |
|  |  | High | -0.138* | 0.002 | -0.238 | -0.039 |
|  | Medium | High | -0.090 | 0.099 | -0.190 | 0.010 |
| 8w | Control | Low | -1.508* | 0.000 | -1.608 | -1.409 |
|  |  | Medium | -1.007* | 0.000 | -1.106 | -0.907 |
|  |  | High | -1.195* | 0.000 | -1.295 | -1.095 |
|  | Low | Medium | 0.502* | 0.000 | 0.402 | 0.601 |
|  |  | High | 0.313* | 0.000 | 0.214 | 0.413 |
|  | Medium | High | -0.188* | 0.000 | -0.288 | -0.089 |
| 12w | Control | Low | -1.695* | 0.000 | -1.795 | -1.595 |
|  |  | Medium | -0.598* | 0.000 | -0.698 | -0.499 |
|  |  | High | -0.398* | 0.000 | -0.498 | -0.299 |
|  | Low | Medium | 1.097* | 0.000 | 0.997 | 1.196 |
|  |  | High | 1.297* | 0.000 | 1.197 | 1.396 |
|  | Medium | High | 0.200* | 0.000 | 0.100 | 0.300 |
| 18w | Control | Long-High | -2.025* | 0.000 | -2.099 | -1.951 |
| #Intermuscular vascular density (Fold of control) in CD34 immunohistochemistry. | | | | | | |
| *The mean difference is significant at the 0.05 level. | | | | | | |

| **Table S18. Simple effect analysis of pairwise comparisons between group and time in vessels of the epimysium** | | | | | | |
| --- | --- | --- | --- | --- | --- | --- |
| **Vascular density^#^** | | | **Mean Difference** | ***P*** | **95% Confidence Interval for Difference** | |
| ***Group*** | ***Time (I)*** | ***Time (J)*** | **(I－J)** |  | **Lower Bound** | **Upper Bound** |
| Control | 6w | 8w | -8.882×10^-16^ | 1.000 | -0.080 | 0.080 |
|  |  | 12w | -3.109×10^-15^ | 1.000 | -0.080 | 0.080 |
|  |  | 18w | 0.000 | 1.000 | -0.080 | 0.080 |
|  | 8w | 12w | -1.785×10^-15^ | 1.000 | -0.080 | 0.080 |
|  |  | 18w | 1.110×10^-15^ | 1.000 | -0.080 | 0.080 |
|  | 12w | 18w | 2.896×10^-15^ | 1.000 | -0.080 | 0.080 |
| Low | 6w | 8w | -0.607* | 0.000 | -0.679 | -0.534 |
|  |  | 12w | -0.030 | 0.680 | -0.103 | 0.043 |
|  | 8w | 12w | 0.577* | 0.000 | 0.504 | 0.649 |
| Medium | 6w | 8w | -1.020* | 0.000 | -1.093 | -0.947 |
|  |  | 12w | -2.420* | 0.000 | -2.493 | -2.347 |
|  | 8w | 12w | -1.400* | 0.000 | -1.473 | -1.327 |
| High | 6w | 8w | -0.290* | 0.000 | -0.363 | -0.217 |
|  |  | 12w | -2.183* | 0.000 | -2.256 | -2.111 |
|  | 8w | 12w | -1.893* | 0.000 | -1.966 | -1.821 |
| ***Time*** | ***Group (I)*** | ***Group (J)*** |  |  |  |  |
| 6w | Control | Low | 0.015 | 0.997 | -0.065 | 0.095 |
|  |  | Medium | 0.022 | 0.977 | -0.059 | 0.102 |
|  |  | High | -0.005 | 1.000 | -0.085 | 0.075 |
|  | Low | Medium | 0.007 | 1.000 | -0.074 | 0.087 |
|  |  | High | -0.020 | 0.985 | -0.100 | 0.060 |
|  | Medium | High | -0.027 | 0.939 | -0.107 | 0.054 |
| 8w | Control | Low | -0.592* | 0.000 | -0.672 | -0.511 |
|  |  | Medium | -0.998* | 0.000 | -1.079 | -0.918 |
|  |  | High | -0.295* | 0.000 | -0.375 | -0.215 |
|  | Low | Medium | -0.407* | 0.000 | -0.487 | -0.326 |
|  |  | High | 0.297* | 0.000 | 0.216 | 0.377 |
|  | Medium | High | 0.703* | 0.000 | 0.623 | 0.784 |
| 12w | Control | Low | -0.015 | 0.997 | -0.095 | 0.065 |
|  |  | Medium | -2.398* | 0.000 | -2.479 | -2.318 |
|  |  | High | -2.188* | 0.000 | -2.269 | -2.108 |
|  | Low | Medium | -2.383* | 0.000 | -2.464 | -2.303 |
|  |  | High | -2.173* | 0.000 | -2.254 | -2.093 |
|  | Medium | High | 0.210* | 0.000 | 0.130 | 0.290 |
| 18w | Control | Long-High | 0.305* | 0.000 | 0.246 | 0.364 |
| #Vascular density (Fold of control) of the epimysium in vWF immunohistochemistry. | | | | | | |
| *The mean difference is significant at the 0.05 level. | | | | | | |

| **Table S19. Simple effect analysis of pairwise comparisons between group and time in VEGF immunohistochemistry** | | | | | | |
| --- | --- | --- | --- | --- | --- | --- |
| **AOD^#^** | | | **Mean Difference** | ***P*** | **95% Confidence Interval for Difference** | |
| ***Group*** | ***Time (I)*** | ***Time (J)*** | **(I－J)** |  | **Lower Bound** | **Upper Bound** |
| Control | 6w | 8w | 0.000 | 1.000 | -0.006 | 0.006 |
|  |  | 12w | -5.000×10^-5^ | 1.000 | -0.006 | 0.006 |
|  |  | 18w | 0.000 | 1.000 | -0.006 | 0.006 |
|  | 8w | 12w | 0.000 | 1.000 | -0.006 | 0.006 |
|  |  | 18w | 0.000 | 1.000 | -0.006 | 0.005 |
|  | 12w | 18w | 0.000 | 1.000 | -0.006 | 0.006 |
| Low | 6w | 8w | 0.003 | 0.392 | -0.002 | 0.008 |
|  |  | 12w | 0.010* | 0.000 | 0.005 | 0.016 |
|  | 8w | 12w | 0.007* | 0.003 | 0.002 | 0.013 |
| Medium | 6w | 8w | -0.019* | 0.000 | -0.024 | -0.014 |
|  |  | 12w | -0.046* | 0.000 | -0.051 | -0.041 |
|  | 8w | 12w | -0.027* | 0.000 | -0.032 | -0.022 |
| High | 6w | 8w | -0.039* | 0.000 | -0.044 | -0.033 |
|  |  | 12w | 0.017* | 0.000 | 0.012 | 0.022 |
|  | 8w | 12w | 0.056* | 0.000 | 0.050 | 0.061 |
| ***Time*** | ***Group (I)*** | ***Group (J)*** |  |  |  |  |
| 6w | Control | Low | -0.025* | 0.000 | -0.031 | -0.019 |
|  |  | Medium | -0.014* | 0.000 | -0.020 | -0.009 |
|  |  | High | -0.017* | 0.000 | -0.023 | -0.011 |
|  | Low | Medium | 0.010* | 0.000 | 0.005 | 0.016 |
|  |  | High | 0.008* | 0.002 | 0.002 | 0.014 |
|  | Medium | High | -0.002 | 0.823 | -0.008 | 0.003 |
| 8w | Control | Low | -0.022* | 0.000 | -0.028 | -0.016 |
|  |  | Medium | -0.034* | 0.000 | -0.039 | -0.028 |
|  |  | High | -0.056* | 0.000 | -0.062 | -0.050 |
|  | Low | Medium | -0.012* | 0.000 | -0.017 | -0.006 |
|  |  | High | -0.034* | 0.000 | -0.040 | -0.028 |
|  | Medium | High | -0.022* | 0.000 | -0.028 | -0.016 |
| 12w | Control | Low | -0.014* | 0.000 | -0.020 | -0.009 |
|  |  | Medium | -0.061* | 0.000 | -0.066 | -0.055 |
|  |  | High | 0.000 | 1.000 | -0.006 | 0.006 |
|  | Low | Medium | -0.046* | 0.000 | -0.052 | -0.040 |
|  |  | High | 0.015* | 0.000 | 0.009 | 0.020 |
|  | Medium | High | 0.061* | 0.000 | 0.055 | 0.067 |
| 18w | Control | Long-High | 0.002 | 0.395 | -0.002 | 0.006 |
| #AOD: Average optical density; VEGF: Vascular endothelial growth factor. | | | | | | |
| *The mean difference is significant at the 0.05 level. | | | | | | |

| **Table S20. Simple effect analysis of pairwise comparisons between group and time in sFRP2 immunohistochemistry** | | | | | | |
| --- | --- | --- | --- | --- | --- | --- |
| **AOD^#^** | | | **Mean Difference** | ***P*** | **95% Confidence Interval for Difference** | |
| ***Group*** | ***Time (I)*** | ***Time (J)*** | **(I－J)** |  | **Lower Bound** | **Upper Bound** |
| Control | 6w | 8w | 0.002 | 0.999 | -0.009 | 0.012 |
|  |  | 12w | 0.001 | 1.000 | -0.009 | 0.011 |
|  |  | 18w | 0.004 | 0.857 | -0.006 | 0.014 |
|  | 8w | 12w | -0.001 | 1.000 | -0.011 | 0.009 |
|  |  | 18w | 0.003 | 0.987 | -0.008 | 0.013 |
|  | 12w | 18w | 0.003 | 0.945 | -0.007 | 0.014 |
| Low | 6w | 8w | 0.010* | 0.035 | 0.001 | 0.019 |
|  |  | 12w | 0.025* | 0.000 | 0.016 | 0.034 |
|  | 8w | 12w | 0.015* | 0.000 | 0.006 | 0.024 |
| Medium | 6w | 8w | -0.004 | 0.681 | -0.013 | 0.005 |
|  |  | 12w | 0.011* | 0.015 | 0.002 | 0.020 |
|  | 8w | 12w | 0.015* | 0.001 | 0.006 | 0.024 |
| High | 6w | 8w | -0.004 | 0.681 | -0.013 | 0.005 |
|  |  | 12w | -0.020* | 0.000 | -0.030 | -0.011 |
|  | 8w | 12w | -0.017* | 0.000 | -0.026 | -0.007 |
| ***Time*** | ***Group (I)*** | ***Group (J)*** |  |  |  |  |
| 6w | Control | Low | -0.042* | 0.000 | -0.052 | -0.032 |
|  |  | Medium | -0.032* | 0.000 | -0.042 | -0.022 |
|  |  | High | -0.045* | 0.000 | -0.055 | -0.035 |
|  | Low | Medium | 0.010 | 0.068 | 0.000 | 0.020 |
|  |  | High | -0.003 | 0.967 | -0.013 | 0.007 |
|  | Medium | High | -0.013* | 0.007 | -0.023 | -0.003 |
| 8w | Control | Low | -0.034* | 0.000 | -0.044 | -0.023 |
|  |  | Medium | -0.037* | 0.000 | -0.048 | -0.027 |
|  |  | High | -0.050* | 0.000 | -0.061 | -0.040 |
|  | Low | Medium | -0.004 | 0.898 | -0.014 | 0.006 |
|  |  | High | -0.017* | 0.000 | -0.027 | -0.006 |
|  | Medium | High | -0.013* | 0.007 | -0.023 | -0.003 |
| 12w | Control | Low | -0.018* | 0.000 | -0.028 | -0.007 |
|  |  | Medium | -0.022* | 0.000 | -0.032 | -0.012 |
|  |  | High | -0.066* | 0.000 | -0.076 | -0.056 |
|  | Low | Medium | -0.004 | 0.857 | -0.014 | 0.006 |
|  |  | High | -0.048* | 0.000 | -0.059 | -0.038 |
|  | Medium | High | -0.044* | 0.000 | -0.054 | -0.034 |
| 18w | Control | Long-High | -0.080* | 0.000 | -0.087 | -0.072 |
| #AOD: Average optical density; sFRP2: secreted frizzled-related protein 2. | | | | | | |
| *The mean difference is significant at the 0.05 level. | | | | | | |

| **Table S21. Simple effect analysis of pairwise comparisons between group and time in YAP1 immunohistochemistry** | | | | | | |
| --- | --- | --- | --- | --- | --- | --- |
| **AOD^#^** | | | **Mean Difference** | ***P*** | **95% Confidence Interval for Difference** | |
| ***Group*** | ***Time (I)*** | ***Time (J)*** | **(I－J)** |  | **Lower Bound** | **Upper Bound** |
| Control | 6w | 8w | -0.003 | 0.893 | -0.010 | 0.004 |
|  |  | 12w | 0.000 | 1.000 | -0.007 | 0.007 |
|  |  | 18w | 0.001 | 1.000 | -0.006 | 0.008 |
|  | 8w | 12w | 0.002 | 0.919 | -0.005 | 0.010 |
|  |  | 18w | 0.003 | 0.706 | -0.004 | 0.011 |
|  | 12w | 18w | 0.001 | 0.999 | -0.006 | 0.008 |
| Low | 6w | 8w | -0.011* | 0.000 | -0.017 | -0.004 |
|  |  | 12w | -0.003 | 0.630 | -0.009 | 0.004 |
|  | 8w | 12w | 0.008* | 0.011 | 0.001 | 0.014 |
| Medium | 6w | 8w | -0.014* | 0.000 | -0.020 | -0.007 |
|  |  | 12w | -0.036* | 0.000 | -0.042 | -0.029 |
|  | 8w | 12w | -0.022* | 0.000 | -0.028 | -0.016 |
| High | 6w | 8w | -0.010* | 0.001 | -0.017 | -0.004 |
|  |  | 12w | 0.003 | 0.543 | -0.003 | 0.010 |
|  | 8w | 12w | 0.013* | 0.000 | 0.007 | 0.020 |
| ***Time*** | ***Group (I)*** | ***Group (J)*** |  |  |  |  |
| 6w | Control | Low | -0.020* | 0.000 | -0.027 | -0.013 |
|  |  | Medium | -0.020* | 0.000 | -0.027 | -0.013 |
|  |  | High | -0.038* | 0.000 | -0.045 | -0.031 |
|  | Low | Medium | 0.000 | 1.000 | -0.007 | 0.008 |
|  |  | High | -0.018* | 0.000 | -0.025 | -0.011 |
|  | Medium | High | -0.018* | 0.000 | -0.025 | -0.011 |
| 8w | Control | Low | -0.028* | 0.000 | -0.035 | -0.021 |
|  |  | Medium | -0.031* | 0.000 | -0.038 | -0.024 |
|  |  | High | -0.046* | 0.000 | -0.053 | -0.039 |
|  | Low | Medium | -0.003 | 0.919 | -0.010 | 0.005 |
|  |  | High | -0.018* | 0.000 | -0.025 | -0.010 |
|  | Medium | High | -0.015* | 0.000 | -0.022 | -0.008 |
| 12w | Control | Low | -0.023* | 0.000 | -0.030 | -0.016 |
|  |  | Medium | -0.055* | 0.000 | -0.062 | -0.048 |
|  |  | High | -0.035* | 0.000 | -0.042 | -0.028 |
|  | Low | Medium | -0.032* | 0.000 | -0.039 | -0.025 |
|  |  | High | -0.012* | 0.000 | -0.019 | -0.005 |
|  | Medium | High | 0.020* | 0.000 | 0.013 | 0.028 |
| 18w | Control | Long-High | 0.011* | 0.000 | 0.006 | 0.016 |
| #AOD: Average optical density; YAP1: Yes-associated protein 1. | | | | | | |
| *The mean difference is significant at the 0.05 level. | | | | | | |

| **Table S22. Simple effect analysis of pairwise comparisons between group and time in p-YAP^S127^ immunohistochemistry** | | | | | | |
| --- | --- | --- | --- | --- | --- | --- |
| **AOD^#^** | | | **Mean Difference** | ***P*** | **95% Confidence Interval for Difference** | |
| ***Group*** | ***Time (I)*** | ***Time (J)*** | **(I－J)** |  | **Lower Bound** | **Upper Bound** |
| Control | 6w | 8w | 1.000×10^-4^ | 1.000 | -0.002 | 0.002 |
|  |  | 12w | 0.000 | 0.994 | -0.001 | 0.002 |
|  |  | 18w | 0.000 | 1.000 | -0.002 | 0.001 |
|  | 8w | 12w | 0.000 | 0.999 | -0.001 | 0.002 |
|  |  | 18w | 0.000 | 0.999 | -0.002 | 0.001 |
|  | 12w | 18w | 0.000 | 0.972 | -0.002 | 0.001 |
| Low | 6w | 8w | -0.002* | 0.009 | -0.003 | 0.000 |
|  |  | 12w | 0.000 | 0.877 | -0.001 | 0.002 |
|  | 8w | 12w | 0.002* | 0.001 | 0.001 | 0.004 |
| Medium | 6w | 8w | 0.024* | 0.000 | 0.022 | 0.025 |
|  |  | 12w | 0.027* | 0.000 | 0.025 | 0.028 |
|  | 8w | 12w | 0.003* | 0.000 | 0.001 | 0.004 |
| High | 6w | 8w | 0.000 | 0.982 | -0.001 | 0.002 |
|  |  | 12w | -0.062* | 0.000 | -0.064 | -0.061 |
|  | 8w | 12w | -0.062* | 0.000 | -0.064 | -0.061 |
| ***Time*** | ***Group (I)*** | ***Group (J)*** |  |  |  |  |
| 6w | Control | Low | 0.001 | 0.556 | -0.001 | 0.003 |
|  |  | Medium | -0.028* | 0.000 | -0.029 | -0.026 |
|  |  | High | 0.000 | 0.972 | -0.001 | 0.002 |
|  | Low | Medium | -0.029* | 0.000 | -0.030 | -0.027 |
|  |  | High | 0.000 | 0.967 | -0.002 | 0.001 |
|  | Medium | High | 0.028* | 0.000 | 0.026 | 0.030 |
| 8w | Control | Low | -0.001 | 0.454 | -0.003 | 0.001 |
|  |  | Medium | -0.004* | 0.000 | -0.006 | -0.002 |
|  |  | High | 0.001 | 0.929 | -0.001 | 0.002 |
|  | Low | Medium | -0.003* | 0.000 | -0.005 | -0.001 |
|  |  | High | 0.002 | 0.064 | -5.501×10^-5^ | 0.003 |
|  | Medium | High | 0.005* | 0.000 | 0.003 | 0.006 |
| 12w | Control | Low | 0.001 | 0.474 | -0.001 | 0.003 |
|  |  | Medium | -0.001 | 0.112 | -0.003 | 0.000 |
|  |  | High | -0.062* | 0.000 | -0.064 | -0.060 |
|  | Low | Medium | -0.002* | 0.001 | -0.004 | -0.001 |
|  |  | High | -0.063* | 0.000 | -0.065 | -0.061 |
|  | Medium | High | -0.061* | 0.000 | -0.062 | -0.059 |
| 18w | Control | Long-High | 0.002* | 0.009 | 0.000 | 0.003 |
| #AOD: Average optical density; p-YAP^S127^: Phospho-YAP1-S127. | | | | | | |
| *The mean difference is significant at the 0.05 level. | | | | | | |

| **Table S23. Simple effect analysis of pairwise comparisons between group and time in TGF-β1 immunofluorescence** | | | | | | |
| --- | --- | --- | --- | --- | --- | --- |
| **TGF-β1^+^ cells%^#^** | | | **Mean Difference** | ***P*** | **95% Confidence Interval for Difference** | |
| ***Group*** | ***Time (I)*** | ***Time (J)*** | **(I－J)** |  | **Lower Bound** | **Upper Bound** |
| Control | 6w | 8w | -0.297 | 1.000 | -3.441 | 2.847 |
|  |  | 12w | 1.671 | 0.635 | -1.473 | 4.816 |
|  |  | 18w | 0.903 | 0.969 | -2.242 | 4.047 |
|  | 8w | 12w | 1.968 | 0.449 | -1.176 | 5.113 |
|  |  | 18w | 1.200 | 0.887 | -1.945 | 4.344 |
|  | 12w | 18w | -0.769 | 0.986 | -3.913 | 2.376 |
| Low | 6w | 8w | 5.641* | 0.000 | 2.800 | 8.482 |
|  |  | 12w | 20.541* | 0.000 | 17.700 | 23.382 |
|  | 8w | 12w | 14.900* | 0.000 | 12.059 | 17.741 |
| Medium | 6w | 8w | -1.065 | 0.741 | -3.906 | 1.776 |
|  |  | 12w | 10.793* | 0.000 | 7.952 | 13.634 |
|  | 8w | 12w | 11.858* | 0.000 | 9.017 | 14.700 |
| High | 6w | 8w | -27.282* | 0.000 | -30.123 | -24.441 |
|  |  | 12w | 5.275* | 0.000 | 2.434 | 8.116 |
|  | 8w | 12w | 32.557* | 0.000 | 29.716 | 35.398 |
| ***Time*** | ***Group (I)*** | ***Group (J)*** |  |  |  |  |
| 6w | Control | Low | 28.407* | 0.000 | 25.263 | 31.552 |
|  |  | Medium | 38.709* | 0.000 | 35.565 | 41.854 |
|  |  | High | 48.039* | 0.000 | 44.895 | 51.184 |
|  | Low | Medium | 10.302* | 0.000 | 7.157 | 13.446 |
|  |  | High | 19.632* | 0.000 | 16.487 | 22.776 |
|  | Medium | High | 9.330* | 0.000 | 6.186 | 12.475 |
| 8w | Control | Low | 34.346* | 0.000 | 31.201 | 37.490 |
|  |  | Medium | 37.941* | 0.000 | 34.797 | 41.085 |
|  |  | High | 21.054* | 0.000 | 17.910 | 24.199 |
|  | Low | Medium | 3.595* | 0.017 | 0.451 | 6.740 |
|  |  | High | -13.292* | 0.000 | -16.436 | -10.147 |
|  | Medium | High | -16.887* | 0.000 | -20.031 | -13.742 |
| 12w | Control | Low | 47.277* | 0.000 | 44.133 | 50.422 |
|  |  | Medium | 47.831* | 0.000 | 44.687 | 50.976 |
|  |  | High | 51.643* | 0.000 | 48.498 | 54.787 |
|  | Low | Medium | 0.554 | 0.998 | -2.590 | 3.698 |
|  |  | High | 4.366* | 0.002 | 1.221 | 7.510 |
|  | Medium | High | 3.811* | 0.010 | 0.667 | 6.956 |
| 18w | Control | Long-High | 17.096* | 0.000 | 14.779 | 19.412 |
| #Percentage of TGF-β1^+^ cells in immunofluorescence staining. | | | | | | |
| *The mean difference is significant at the 0.05 level. | | | | | | |

| **Table S24. Simple effect analysis of pairwise comparisons between group and time in FHL2 immunohistochemistry** | | | | | | |
| --- | --- | --- | --- | --- | --- | --- |
| **AOD^#^** | | | **Mean Difference** | ***P*** | **95% Confidence Interval for Difference** | |
| ***Group*** | ***Time (I)*** | ***Time (J)*** | **(I－J)** |  | **Lower Bound** | **Upper Bound** |
| Control | 6w | 8w | 0.003 | 0.957 | -0.006 | 0.011 |
|  |  | 12w | 0.001 | 0.997 | -0.007 | 0.010 |
|  |  | 18w | 0.000 | 1.000 | -0.008 | 0.008 |
|  | 8w | 12w | -0.001 | 1.000 | -0.009 | 0.007 |
|  |  | 18w | -0.003 | 0.942 | -0.011 | 0.005 |
|  | 12w | 18w | -0.002 | 0.995 | -0.010 | 0.006 |
| Low | 6w | 8w | -0.045* | 0.000 | -0.052 | -0.037 |
|  |  | 12w | -0.039* | 0.000 | -0.047 | -0.032 |
|  | 8w | 12w | 0.005 | 0.199 | -0.002 | 0.013 |
| Medium | 6w | 8w | 0.006 | 0.142 | -0.001 | 0.013 |
|  |  | 12w | -0.022* | 0.000 | -0.030 | -0.015 |
|  | 8w | 12w | -0.028* | 0.000 | -0.036 | -0.021 |
| High | 6w | 8w | -0.043* | 0.000 | -0.051 | -0.036 |
|  |  | 12w | -0.026* | 0.000 | -0.033 | -0.018 |
|  | 8w | 12w | 0.018* | 0.000 | 0.010 | 0.025 |
| ***Time*** | ***Group (I)*** | ***Group (J)*** |  |  |  |  |
| 6w | Control | Low | 0.068* | 0.000 | 0.060 | 0.076 |
|  |  | Medium | 0.035* | 0.000 | 0.027 | 0.043 |
|  |  | High | 0.046* | 0.000 | 0.038 | 0.054 |
|  | Low | Medium | -0.033* | 0.000 | -0.041 | -0.025 |
|  |  | High | -0.022* | 0.000 | -0.030 | -0.014 |
|  | Medium | High | 0.010* | 0.005 | 0.002 | 0.019 |
| 8w | Control | Low | 0.021* | 0.000 | 0.013 | 0.029 |
|  |  | Medium | 0.039* | 0.000 | 0.031 | 0.047 |
|  |  | High | 0.000 | 1.000 | -0.008 | 0.008 |
|  | Low | Medium | 0.018* | 0.000 | 0.010 | 0.026 |
|  |  | High | -0.021* | 0.000 | -0.029 | -0.012 |
|  | Medium | High | -0.039* | 0.000 | -0.047 | -0.031 |
| 12w | Control | Low | 0.027* | 0.000 | 0.019 | 0.035 |
|  |  | Medium | 0.012* | 0.001 | 0.004 | 0.020 |
|  |  | High | 0.019* | 0.000 | 0.011 | 0.027 |
|  | Low | Medium | -0.015* | 0.000 | -0.024 | -0.007 |
|  |  | High | -0.008* | 0.036 | -0.017 | 0.000 |
|  | Medium | High | 0.007 | 0.129 | -0.001 | 0.015 |
| 18w | Control | Long-High | 0.079* | 0.000 | 0.073 | 0.085 |
| #AOD: Average optical density; FHL2: Four and a half LIM domains protein 2. | | | | | | |
| *The mean difference is significant at the 0.05 level. | | | | | | |
